# IRE1α-XBP1s pathway promotes prostate cancer by activating c-MYC signaling

**DOI:** 10.1038/s41467-018-08152-3

**Published:** 2019-01-24

**Authors:** Xia Sheng, Hatice Zeynep Nenseth, Su Qu, Omer F. Kuzu, Turid Frahnow, Lukas Simon, Stephanie Greene, Qingping Zeng, Ladan Fazli, Paul S. Rennie, Ian G. Mills, Håvard Danielsen, Fabian Theis, John B. Patterson, Yang Jin, Fahri Saatcioglu

**Affiliations:** 1 Department of Biosciences, University of Oslo, 0316 Oslo, Norway; 2 School of Public Health, Tongji Medical College, Huazhong University of Science and Technology, 430030 Wuhan, China; 3 Institute of Computational Biology, Helmholtz Zentrum München, 85764 Neuherberg, Germany; 4 Faculty of Business Administration and Economics, Chair DataScience, University Bielefeld, 33615 Bielefeld, Germany; 5 Fosun Orinove, Inc., Unit 211, Building A4, 218 Xinhu Street, 215000 SuZhou, China; 6 The Vancouver Prostate Centre, Vancouver, BC V6H3Z6 Canada; 7 Movember/PCUK Centre of Excellence for Prostate Cancer Research, Centre for Cancer Research and Cell Biology (CCRCB), Queen’s University of Belfast, Belfast, BT7 1NN UK; 8 Institute for Cancer Genetics and Informatics, Oslo University Hospital, 0379 Oslo, Norway; 9 Center for Cancer Biomedicine, University of Oslo, 0316 Oslo, Norway; 10 Department of Informatics, University of Oslo, 0316 Oslo, Norway; 11 Nuffield Division of Clinical Laboratory Sciences, University of Oxford, Oxford, OX3 7LF UK

**Keywords:** Prostate cancer, Stress signalling, Drug discovery, Transcriptomics

## Abstract

Activation of endoplasmic reticulum (ER) stress/the unfolded protein response (UPR) has been linked to cancer, but the molecular mechanisms are poorly understood and there is a paucity of reagents to translate this for cancer therapy. Here, we report that an IRE1α RNase-specific inhibitor, MKC8866, strongly inhibits prostate cancer (PCa) tumor growth as monotherapy in multiple preclinical models in mice and shows synergistic antitumor effects with current PCa drugs. Interestingly, global transcriptomic analysis reveal that IRE1α-XBP1s pathway activity is required for c-MYC signaling, one of the most highly activated oncogenic pathways in PCa. XBP1s is necessary for optimal c-MYC mRNA and protein expression, establishing, for the first time, a direct link between UPR and oncogene activation. In addition, an XBP1-specific gene expression signature is strongly associated with PCa prognosis. Our data establish IRE1α-XBP1s signaling as a central pathway in PCa and indicate that its targeting may offer novel treatment strategies.

## Abstract (web summary)

ER stress and UPR are implicated in various cancers. Here, the authors show that one of the canonical UPR pathways, IRE1α-XBP1 regulates c-MYC signaling to promote prostate tumorigenesis, and pharmacological inhibition of IRE1α with MKC8866 inhibits prostate cancer growth and synergizes with clinically used prostate cancer drugs.

## Introduction

During cancer development, there is a need for significantly increased protein synthesis to support heightened proliferation and migration. In addition, there is limited oxygen and nutrient supply to the growing tumor. These events, among other pathways, and similar to in normal cells, result in endoplasmic reticulum (ER) stress and activate the unfolded protein response (UPR) in an attempt to resolve these stresses to enable survival^1,2^. If the insult is not resolved, however, prolonged ER stress and UPR activation initiates mechanisms of cell death.

Previous studies have implicated UPR activation in different aspects of carcinogenesis and a variety of cancer types^3–5^. A number of small molecule drugs were recently identified to interfere with various arms of the UPR^6^. However, potential translation of this body of knowledge to cancer therapy has been limited to date.

Recent work has shown that UPR has a key role in prostate cancer (PCa)^7^, the most commonly diagnosed non-skin cancer and the second most deadly cancer in Western countries^8^. PCa is initially hormone-dependent for growth, which is the basis for androgen deprivation therapy (ADT); however, most tumors eventually relapse to castration resistant PCa (CRPC)^9^. Recently developed second generation antiandrogens, such as abiraterone and enzalutamide, as well as specific chemotherapies, such as taxanes, have efficacy treating this lethal stage of the disease^10^. However, inherent or acquired resistance to these agents remain major clinical challenges, which makes it imperative to explore novel therapeutic approaches.

One of the canonical transmembrane sensors of UPR is inositol requiring-enzyme 1 alpha (IRE1α). Upon activation, IRE1α cleaves the X-box-binding protein 1 (*XBP1*) mRNA into a spliced form that encodes an active transcription factor (XBP1s), which then activates expression of UPR target genes. We recently found that androgen signaling, the most central signaling pathway in PCa, activates the IRE1α arm of UPR^11^. Consistent with the importance of this regulation, genetic inhibition of IRE1α or XBP1 interferes with PCa growth in vitro and in vivo^11^.

In the present study, we used an optimized IRE1α RNase-specific inhibitor, MKC8866, and found that it strongly inhibited PCa growth in multiple preclinical models in vitro and in vivo. MKC8866 also had robust synergistic effects in combination with clinical agents. Critically, RNA sequencing (RNA-seq) analysis of MKC8866-treated or *XBP1* knockdown PCa cells revealed that IRE1α-XBP1s is essential for c-MYC signaling, a central oncogenic regulatory pathway in PCa^12,13^. These results establish IRE1α-XBP1s signaling as a potential target for alternative treatment strategies for PCa.

## Results

### AR and UPR gene expression are correlated in CRPC

We previously showed that androgen receptor (AR) activated the IRE1α-XBP1s signaling in LNCaP and VCaP cells that model the androgen-sensitive state of PCa^11^. To assess whether this applies to CRPC, we used two established CRPC models, 22Rv1 and C4-2B lines, both of which are responsive to, but not dependent on, androgens. Androgens significantly upregulated IRE1α and XBP1s expression, as well as that of XBP1s target P58IPK in both cell lines (Supplementary Fig. 1a). Consistently, there was a significant positive correlation between AR gene expression signature and IRE1 arm gene expression in four independent gene expression datasets from patients with both primary and metastatic PCa, including CRPC (Supplementary Fig. 1b). These data suggest that androgens are important for IRE1α-XBP1s arm activation in all phases of PCa.

### Discovery and characterization of an optimized specific IRE1α inhibitor−MKC8866

**Fig. 1:**
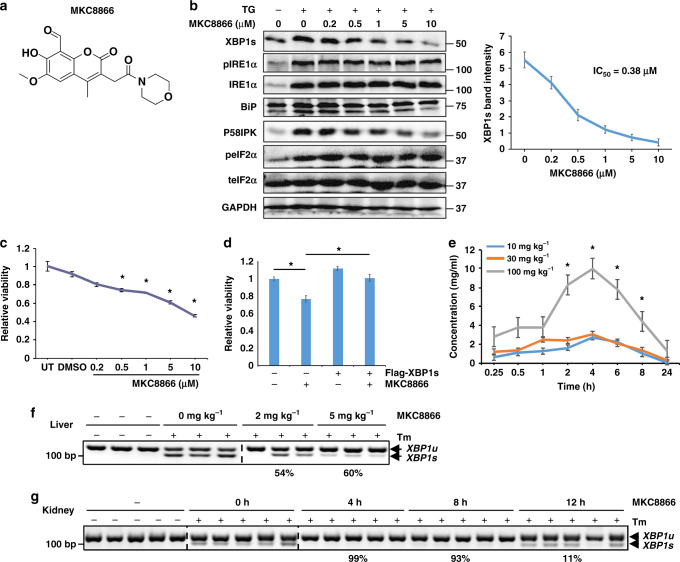
Functional characterization of MKC8866 on IRE1α RNase activity. **a** Chemical structure of MKC8866. **b** LNCaP cells were cultured in regular growth medium and treated with 30 nM TG and the indicated doses of MKC8866 for 24 h. Protein expression was determined by Western analysis. XBP1s Western blot intensity was quantified and normalized to GAPDH from three different experiments, and used to calculate the IC_50_. **c** LNCaP cells were either left untreated (UT), or treated with vehicle (DMSO) or MKC8866 with indicated doses. Cell viability was measured after 3 days. **d** LNCaP cells were transfected with either empty vector (pCDNA3) or the pCDNA3-Flag-XBP1s plasmid, and treated with vehicle (DMSO) or 10 μM MKC8866. Cell viability was measured after 3 days. **e** Mice (*n* = 3) were orally dosed with indicated amounts of MKC8866 and its plasma concentration was profiled post treatment. **f** Mice (*n* = 3) were intraperitoneally injected with 1 mg kg^−1^ tunicamycin (Tm) for 4 h and orally gavaged with the indicated dosage of MKC8866 for 2 h before harvesting its liver. Quantified average inhibition ratio is shown below the corresponding treatment. **g** Mice (*n* = 5) were dosed with 1 mg kg^−1^ tunicamycin (Tm) and 4 mg kg^−1^ MKC8866 for the indicated time length before harvesting its kidney. Quantified average inhibition ratio is shown below the corresponding treatment. *P < 0.05, Student *t*-test, error bars denote SD

MKC8866 (see Fig. 1a for chemical structure) was optimized and refined from a family of IRE1α-specific endoribonuclease inhibitors obtained from a chemical library screen^14–16^. Previous characterization of these compounds, including structural analyses, confirmed its specificity on IRE1α RNase activity^15^. Similar to its earlier versions, MKC8866 potently inhibited the RNase activity of human IRE1α in vitro with an IC_50_ of 0.29 μM (Supplementary Fig. 2a). In MM1 myeloma cells, MKC8866 strongly inhibited DTT-induced XBP1s expression with an EC_50_ of 0.52 μM (Supplementary Fig. 2b). Dose titration experiments in unstressed RPMI 8226 cells corroborated these data with an IC_50_ of 0.14 μM (Supplementary Fig. 2c).

In LNCaP PCa cells, MKC8866 suppressed XBP1s expression in a dose-dependent manner under conditions of mild ER stress (30 nM thapsigargin, TG) with an IC_50_ of 0.38 μM (Fig. 1b). The expression of two XBP1s target genes, BiP and P58IPK, were similarly inhibited, whereas phospho-IRE1α or total IRE1α, or phospho-eIF2α, levels were not affected (Fig. 1b). Similar results were obtained in three independent PCa cell lines (VCaP, 22Rv1, and C4-2B) modeling different stages of PCa (Supplementary Fig. 2d). MKC8866 suppressed the viability of all four cell lines in a dose-dependent manner under normal conditions, with the most robust effect in LNCaP cells (Fig. 1c and Supplementary Fig. 2e). The efficacy of MKC8866 was further increased in C4-2B cells under TG-induced mild ER stress (Supplementary Fig. 2f). Ectopically expressed XBP1s rescued the inhibitory effect of MKC8866 (Fig. 1d). The levels of ectopically expressed flag-XBP1s was a few-fold higher compared with endogenous XPB1s; whereas endogenous XBP1s expression was inhibited by MKC8866, that of flag-XBP1s was not affected (Supplementary Fig. 2g). Furthermore, MKC8866 potently impaired XBP1s levels induced either by androgen treatment (Supplementary Fig. 2h), or glucose deprivation (Supplementary Fig. 2i). In addition, MKC8866 had moderate oral bioavailability (30%) in mice, with maximum concentrations in blood observed at 4 h after oral administration (Fig. 1e); it also suppressed the tunicamycin (Tm)-induced *XBP1* splicing in mouse liver (Fig. 1f), as well as in mouse kidney (Fig. 1g). These data suggest that MKC8866 effectively represses IRE1α-mediated *XBP1* splicing in PCa cells and has favorable pharmacokinetic and pharmacodynamic properties.

### IRE1α targeting inhibits PCa cell growth in vitro and in vivo

We next investigated the potential effect of MKC8866 on PCa cell growth under various culture conditions. MKC8866 significantly reduced colony formation in all four PCa cell lines tested under either anchorage-dependent or anchorage-independent conditions (Supplementary Fig. 3a). Prostatosphere growth of these cell lines, an indication of stemness, was also markedly inhibited in the presence of MKC8866, which was consistent with IRE1α or XBP1 knockdown experiments (Supplementary Fig. 3b), suggesting that IRE1α may be involved in mechanisms of tumor initiation in PCa.

**Fig. 2:**
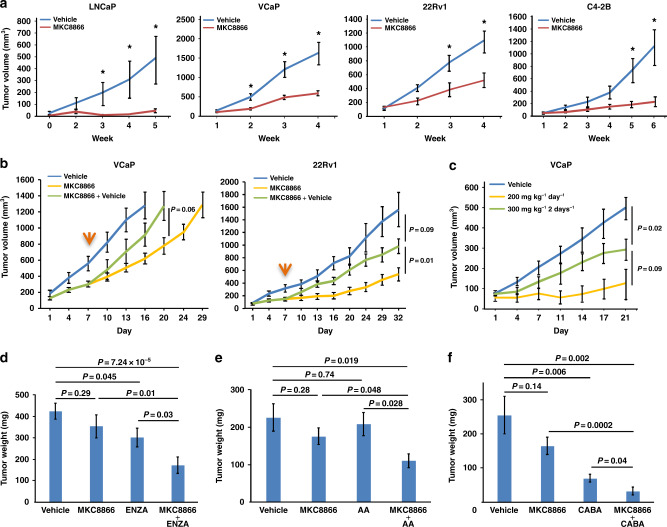
Therapeutic efficacy of MKC8866 in preclinical mouse models of PCa. **a** Nude mice bearing LNCaP, VCaP, 22Rv1, or C4-2B xenografts were treated orally with either vehicle or 300 mg kg^−1^ MKC8866 daily and tumor growth was recorded. **b** Nude mice bearing VCaP or 22Rv1 tumors were treated with either vehicle or 300 mg kg^−1^ MKC8866 daily. At day 7 (indicated by the orange arrow), the MKC8866-treated mice were randomly divided into two groups, one of which continuously received MKC8866 of the same dosage while the other group was treated with vehicle for the rest of the experiment. **c** Nude mice bearing VCaP xenografts were orally treated with either vehicle, 200 mg kg^−1^ MKC8866 daily, or 300 mg kg^−1^ MKC8866 every other day. Tumor growth was recorded weekly. **d** Nude mice bearing VCaP xenografts were orally treated either with vehicle, MKC8866 (300 mg kg^−1^ every two days), enzalutamide (ENZA, 30 mg kg^−1^ every two days), or MKC8866+ENZA. Tumor weight was recorded at the end of the experiment. **e** As in **d**, but the treatments were vehicle, MKC8866 (300 mg kg^−1^ every two days), abiraterone acetate (AA, 20 mg kg^−1^ every two days), or a combination of both drugs. **f** As in **d**, but the treatments were vehicle, or MKC8866 (300 mg kg^−1^ every two days), or cabazitaxel (CABA, 5 mg kg^−1^) twice a week intraperitoneally, or a combination of both drugs. *P < 0.05, all *P* values were calculated by Student’s *t*-test, error bars denote the SEM

Based on these favorable in vitro results, we next evaluated the efficacy of MKC8866 in vivo. Consistent with the in vitro findings, MKC8866 strongly inhibited xenografted tumor growth in all PCa cell lines tested (Fig. 2a). XBP1s expression was significantly lower in MKC8866-treated tumors compared to controls, confirming that MKC8866 was active in mice harboring the tumors and that IRE1α activity was appropriately inhibited in vivo (Supplementary Fig. 4a). In addition, there was a decrease in PCNA expression and an increase in cleaved Caspase-3 levels, indicating that MKC8866 treatment resulted in decreased proliferation and increased apoptosis, respectively (Supplementary Fig. 4b). Removal of MKC8866 during the course of the treatment resulted in rebounding of XBP1s levels and enhanced tumor growth (Fig. 2b and Supplementary Fig. 4c), indicating the importance of sustained MKC8866 application for its growth inhibitory effects. These results show that pharmacological targeting of IRE1α exerts potent antitumor effects in preclinical mouse models of PCa.

### MKC8866 synergizes with clinical PCa drugs in vitro and in vivo

The striking effect of MKC8866 as monotherapy on PCa tumor growth in vivo suggests that it can potentially synergize with drugs that are currently in clinical use for PCa. To assess this, LNCaP cells were treated with sub-optimal doses of MKC8866 along with antiandrogens, including abiraterone acetate and enzalutamide, two drugs that are used in the management of CRPC, as well as taxanes, including docetaxel, cabazitaxel, and paclitaxel. There was additive inhibition on LNCaP cell viability when MKC8866 was combined with either abiraterone acetate, enzalutamide, paclitaxel, or docetaxel, while clear synergy was seen in combination with cabazitaxel (Supplementary Fig. 5a). In CRPC cell lines 22Rv1 and C4-2B, MKC8866 also showed additive effects with abiraterone acetate in vitro, but failed to re-sensitize these cells to enzalutamide (Supplementary Fig. 5b and 5c). These results suggested that there may be enhanced efficacy of currently used PCa drugs when administered in combination with MKC8866 in vivo.

In search for a potentially suitable dose of MKC8866 for an in vivo combination experiment, we reduced its concentration (200 mg kg^−1^) or frequency of its administration (every two days) in a pilot experiment. Daily treatment with the lower dose still strongly inhibited tumor growth, whereas the normal dose every other day was less effective and was thus chosen for further combinatorial tests in vivo (Fig. 2c).

There was strong synergy in tumor growth inhibition when MKC8866 was co-administered with enzalutamide (Fig. 2d). Hematoxylin and eosin (H&E) staining of the tumors revealed that combinatorial treatment had the lowest cell content concomitant with highest necrosis levels among the different treatment regimens demonstrating increased efficacy, which was accompanied by loss of nuclear PCNA expression and marked elevation in caspase-3 staining (Supplementary Fig. 6a–c). Western analysis confirmed reduced expression of XBP1s in tumors treated with both drugs compared to those treated with either vehicle or a single drug, while no significant differences were observed on ATF6α cleavage or eIF2α phosphorylation, indicating that the other canonical UPR arms were not affected (Supplementary Fig. 6d). Co-administration of MKC8866 with abiraterone acetate and cabazitaxel also synergistically inhibited tumor growth (Fig. 2e, f). None of the treatments significantly affected body weight of mice (Supplementary Fig. 6e), suggesting that there were no noteworthy toxicities. Taken together, these data demonstrate that in preclinical models MKC8866 synergizes with some of the central PCa drug regimens that are currently used in the clinic.

### XBP1s is required for activation of the c-MYC transcriptional program

**Fig. 3:**
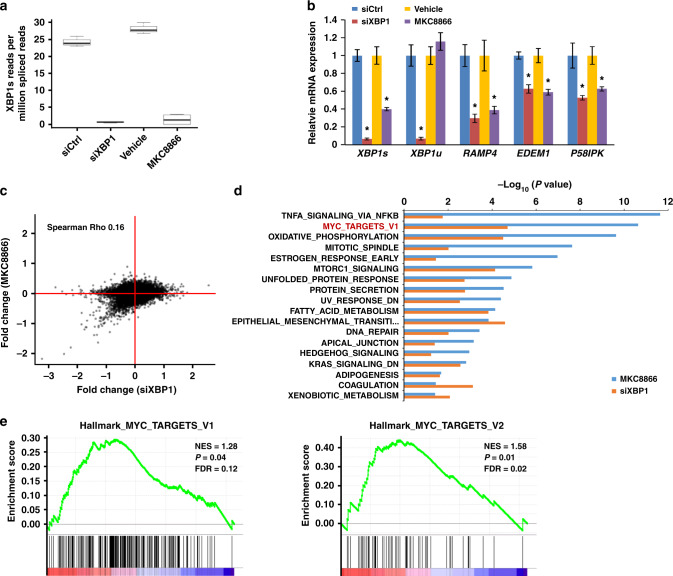
IRE1/XBP1s pathway is required for multiple hallmark pathways in PCa. LNCaP cells were either left untreated or subjected to siRNA-mediated XBP1 knockdown. In parallel, cells were either treated with vehicle or MKC8866. Total RNA was isolated and used in NextGen sequencing. **a** The presence of the XBP1s junction is measured in the spliced RNA-seq reads and represented as a ratio of total spliced reads. The box represents the interquartile range, the horizontal line in box is the median, and the whiskers represent 1.5 times interquartile range. **b** The expression levels of XBP1s, XBP1u, and three XBP1s target genes were determined by qPCR. *P < 0.05, Student *t*-test, error bars denote SD. **c** Scatter plot representing the concordance of gene expression data obtained upon XBP1 knockdown and MKC8866 treatment. **d** Hallmark pathways enriched under both MKC8866-treated and XBP1 knockdown cells. Graph displays category scores as −log_10_ (*P* value) from Fisher’s exact test. **e** Hallmark pathways MYC TARGETS V1 and V2 both enriched by GSEA on combined data of siXBP1 and MKC8866 treatment

MKC8866 specifically inhibits the RNase activity of IRE1α thus decreasing XBP1s levels, which should thus be responsible for the phenotypic effects that we observe in PCa cells. To gain insight into this process and XBP1s-regulated genes, we performed RNA-seq analysis in LNCaP cells upon either *XBP1* siRNA-mediated knockdown (siXBP1) or MKC8866-mediated IRE1α inhibition. Differential gene expression analysis of the RNA-seq data was highly concordant amongst experimental replicates for both siXBP1-treated and MKC8866-treated cells (Supplementary Fig. 7a and 7b). Both approaches robustly depleted *XBP1s* expression, indicated by a significant decrease in the mRNA levels of the spliced compared to the unspliced *XBP1* isoforms (siXBP1: *P* < 2.2e−16, MKC8866: *P* < 2.2e−16, negative binomial generalized linear model) (Fig. 3a). This was further confirmed by qPCR analysis of *XBP1s* mRNA expression, as well as expression of its target genes *RAMP4*, *EDEM1*, and *P58IPK* (Fig. 3b). Interestingly, scatter-plot analysis showed that the down-regulated genes upon XBP1 knockdown and MKC8866 treatment showed high concordance, whereas no strong correlation was observed for the upregulated genes (compare lower left quadrant with other quadrants, Fig. 3c), indicating that XBP1s primarily functions as a transcriptional activator in PCa cells. Consistently, there was significant overlap (26 genes) between the top 100 down-regulated genes (ranked by *P* value) in each treatment (*P* < 2.2e−16, binomial test), whereas the overlap (three genes) was much smaller between the top 100 upregulated genes (*P* < 0.05, binomial test). This suggested that the down-regulated genes by IRE1α inhibition in PCa cells are largely mediated by XBP1s. We also validated the RNA-seq data by individual qPCR analysis of top 10 genes where their expression was inhibited upon both MKC8866 treatment and XBP1 knockdown (Supplementary Fig. 7c).

Hallmark pathway enrichment analysis of the RNA-seq data showed that MKC8866 treatment and *XBP1* knockdown affected similar pathways (Fig. 3d). As expected, UPR and Protein Secretion pathways were both strongly correlated with XBP1 gene expression profile (Supplementary Fig. 8a), mirroring the established central role of IRE1α-XBP1s signaling in these processes^4,17^. Interestingly, among the most highly enriched pathways by both treatments was signaling by c-MYC (ranked first in siXBP1 and second in MKC8866 by *P* value) (Fig. 3d), an oncoprotein which is very frequently deregulated in cancer^12^ and has been centrally implicated in PCa^13^. XBP1-induced gene expression changes were positively associated with two different hallmark signatures for MYC TARGETS, V1 and V2 (Fig. 3e). Furthermore, Kyoto Encyclopedia of Genes and Genome (KEGG) analysis demonstrated that the ribosome pathway ranks at the top in both conditions (Supplementary Fig. 8b and 8c), whereas gene set enrichment analysis (GSEA) revealed XBP1-deregulated genes were positively enriched in KEGG ribosome and protein export (Supplementary Fig. 8d). Together, these data indicate that XBP1 not only affects fundamental aspects of ER biology in PCa cells, consistent with previous findings in other tissues, but also impacts oncogenic c-MYC signaling.

### XBP1s directly activates c-MYC expression

**Fig. 4:**
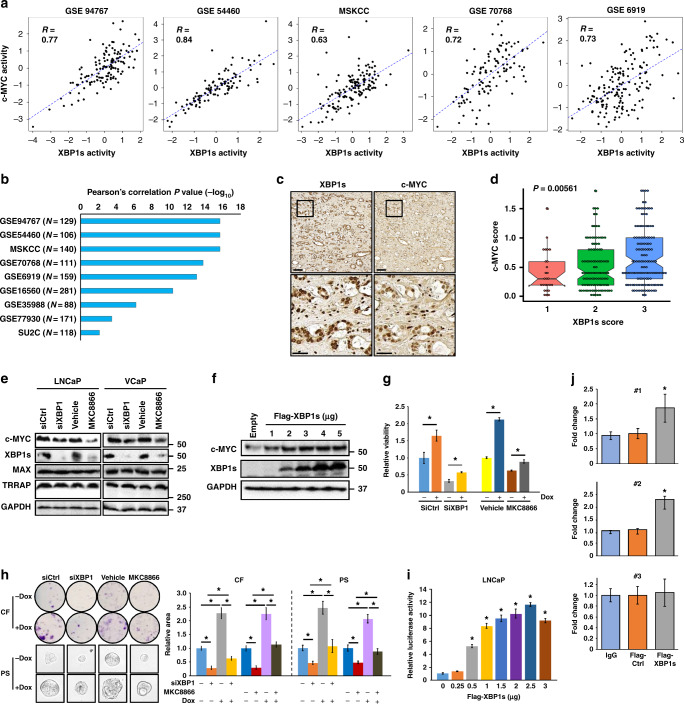
XBP1s activates c-MYC expression and activity in human PCa. **a** The plots show significant positive correlation between XBP1s and c-MYC target gene expression in five independent PCa datasets. **b** The bar graph shows the *P* values of the correlation between XBP1s and c-MYC target gene expression in nine different human PCa cohorts, using the Pearson correlation test. **c** Consecutive sections of human PCa prostatectomy samples were immunostained for XBP1s and c-MYC. Representative images from one patient at two different magnifications are shown. Scale bars, 100 μΜ (upper panel) and 30 μΜ (lower panel). **d** Tissue microarrays containing 260 tumor samples stained with either a XBP1s or c-MYC-specific antiserum were scored. Dots represent values of individual samples; thick horizontal lines represent the median; box represents the upper and lower quartile; whiskers represent 1.5 times interquartile range. The *P* value indicates the significance of correlation between XBP1s and c-MYC scores by ANOVA. **e** LNCaP or VCaP cells were treated with either siXBP1 or MKC8866, and c-MYC levels were determined. **f** LNCaP cells were transfected with either empty vector or flag-XBP1s expression vector. XBP1s and c-MYC levels were then determined after 2 days. **g** LNCaP c-MYC inducible cells were treated with either siXBP1 or MKC8866 and then grown with or without doxycycline (Dox). Cell viability was measured after 3 days. **h** Same procedure was performed as in **g**, colony formation (CF) was measured after 2 weeks while prostatospheres (PS) after 1 week. **i** LNCaP cells were transfected with 1 μg of pGL3-MYC luciferase reporter plasmid plus either empty vector (pCDNA3) or the pCDNA3-Flag-XBP1s plasmid. Luciferase activity was determined after 48 h. Results are from a representative experiment in triplicate. * Indicates statistical differences in LUC values compared to vector-transfected cells (*P* < 0.05) by Student's *t*-test. **j** LNCaP cells were transfected with either empty pCDNA3 vector (Ctrl) or the pCDNA3-Flag-XBP1s plasmid. After 48 h, ChIP assay was performed using Flag antibody. The data are representative of two experiments in duplicate. Two responsive sites (#1 and #2) as well as one non-responsive site (#3) are presented. *P < 0.05, Student's *t*-test, error bars denote the SD

To explore whether XBP1s expression may indeed be linked to c-MYC expression in PCa, we analyzed published gene expression datasets for human PCa. Strikingly, there was a positive and strong correlation between XBP1s and c-MYC target gene expression in 9 out of 11 available cohorts (Fig. 4a, b, and Supplementary Fig. 9a). To assess whether this correlation extends to the protein level, serial sections of human prostatectomy samples were examined by immunohistochemistry for XBP1s and c-MYC. As shown in Fig. 4c, there was clear colocalization of XBP1s and c-MYC expression in human PCa specimens. Furthermore, XBP1s and c-MYC expression was significantly correlated in a tissue microarray (TMA) consisting of 260 human PCa specimens^11,18^ (Fig. 4d). These results suggest that XBP1s expression is functionally linked to c-MYC signaling in human PCa.

One potential mechanism for these observations is that c-MYC expression is regulated by the IRE1α-XBP1s axis. In support of this hypothesis, c-MYC protein expression was inhibited by *XBP1* knockdown as well as MKC8866 treatment, in both LNCaP and VCaP cells, whereas levels of its heterodimer partner MAX and cofactor TRRAP were not significantly affected (Fig. 4e). Consistently, there was a significant decrease in *c-MYC* mRNA expression, as well as its target gene expression, represented by *SHMT1*, *PCNA*, *MTHFD1, CDC25A, ATF4*, and *PPAT*, whereas *MAX* and *TRRAP* mRNA levels were unchanged upon *XBP1* knockdown or MKC8866 treatment (Supplementary Fig. 9b). In contrast, ectopic expression of XBP1s dose-dependently increased c-MYC protein expression in LNCaP (Fig. 4f) and VCaP cells (Supplementary Fig. 9c), which led to an increased resistance to the bromodomain inhibitor JQ1 (Supplementary Fig. 9d). In keeping with this finding, c-MYC immunohistochemical staining intensity was significantly weaker in xenograft tumor sections from MKC8866-treated mice compared with untreated mice (Supplementary Fig. 9e). Furthermore, XBP1-knockdown or MKC8866-mediated inhibition of LNCaP cell viability, colony formation, as well as prostatosphere growth were rescued, at least in part, upon inducible expression of c-MYC (Fig. 4g, h). Similar results were obtained upon c-MYC ectopic expression (Supplementary Fig. 9f and 9g). In addition, ectopic expression of flag-XBP1s activated a *c-MYC* promoter-driven luciferase reporter in a dose-dependent manner in LNCaP (Fig. 4i) and 293T cells (Supplementary Fig. 9h). TG-induced mild stress also activated the *c-MYC* reporter activity in 293T cells, while stronger stress did not, likely due to the translation inhibition by PERK-eIF2α activation (Supplementary Fig. 9i). Finally, chromatin immunoprecipitation (ChIP) analysis showed that XBP1s associated with two response elements in the *c-MYC* gene 5ʹ flanking region (Fig. 4j); consistently, the deletion of these elements in the *c-MYC* promoter significantly impaired the *c-MYC* reporter activation in 293T cells (Supplementary Fig. 9j), indicating that XBP1s directly regulates *c-MYC* transcription. Together, these data suggest that the IRE1α-XBP1s axis is required for c-MYC expression and function in PCa cells.

### XBP1 gene expression signature is strongly associated with PCa prognosis

**Fig. 5:**
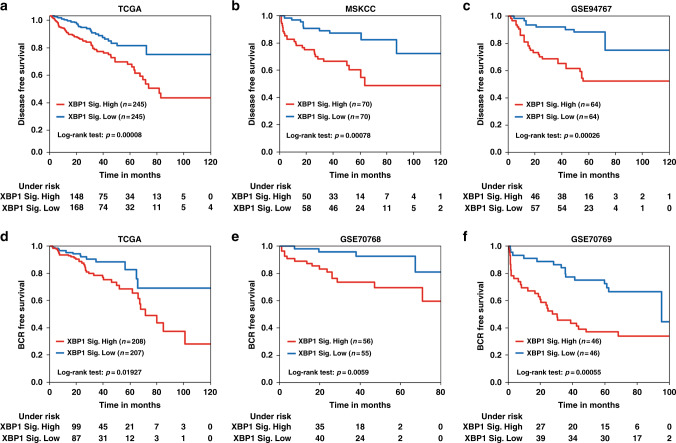
XBP1 gene signature is strongly associated with prostate cancer prognosis. Kaplan–Meier graphs demonstrating a significant association between high expression of the XBP1 signature (red line) and shorter disease-free survival in the TCGA (**a**), MSKCC (**b**) and GSE94767 (**c**) patient datasets. Kaplan–Meier curves showing significant association of elevated XBP1 gene signature expression (red line) with shorter biochemical recurrence (BCR) free survival in TCGA (**d**), GSE70768 (**e**), and GSE70769 (**f**) cohorts of PCa patients. The log-rank test *P* values are shown

Integrated analysis of gene expression profiles upon XBP1 knockdown and MKC8866 treatment identified 733 genes which were significantly downregulated by XBP1 knockdown or MKC8866 (Supplementary Data 1). Next, we evaluated the possibility that the expression of these genes could serve as potential prognostic biomarkers for PCa. To assess this, a subset of significantly differentially expressed IRE1α-XBP1s genes was tested as disease-free survival predictors in three independent cohorts of PCa patients. To avoid cohort-dependent effects, the data was quantile normalized. Eventually, a five-gene combination, defined as the XBP1 signature, was identified using an unbiased prediction and feature selection algorithm for high-dimensional survival regression^19^. This five-gene signature, including *ANLN*, *CSNK1G3*, *RRM2*, *SLC35A2*, and *UBAC2*, demonstrated remarkable predictive power on disease-free survival in the TCGA (logrank test, *P* = 8 × 10^−5^), MSKCC (logrank test, *P* = 7.8 × 10^−4^), and GSE94767 datasets (logrank test, *P* = 2.6 × 10^−4^) (Fig. 5a–c). Further analyses showed significant correlation between elevated XBP1 signature gene expression and shorter biochemical recurrence (BCR)-free survival in the TCGA (logrank test, *P* = 1.9 × 10^−2^), GSE70768 (logrank test, *P* = 5.9 × 10^−3^), and GSE70769 datasets (logrank test, *P* = 5.5 × 10^−4^) (Fig. 5d–f). Taken together, these data show that XBP1 pathway activation correlates with poor survival in PCa patients and a gene signature derived from XBP1-regulated genes may have clinical utility.

## Discussion

Previous work has shown that UPR signaling can affect various aspects of tumor cell biology including angiogenesis, invasion, mitochondrial function, intercellular communication, tumor-associated inflammation, as well as therapy resistance^3–5^. However, potential translational applications for targeting UPR have largely been missing. Here, we have shown that MKC8866, an IRE1α RNase-specific inhibitor molecule, displays significant therapeutic activity in various preclinical models of PCa in vivo.

The major output of IRE1α RNase activity is the synthesis of the transcription factor XBP1s that was previously implicated in cancer. XBP1s is overexpressed in a variety of cancers^7^ suggesting that it acts as a proto-oncogene. XBP1s overexpression in cancer cells can directly promote tumorigenesis, such as in chronic lymphocytic leukemia^20^, and targeting activated IRE1α and subsequent *XBP1* splicing had promising results in preclinical models in multiple myeloma^6^. Furthermore, XBP1s interacts with hypoxia-inducible factor 1-alpha (HIF1α) in triple negative breast cancer and drives tumor progression by inducing a hypoxia signature gene expression program^21^. Interestingly, XBP1 can blunt antitumor activity by interfering with the function of tumor-associated dendritic cells^22^. The XBP1s and ATF6α target, P58IPK, has also been linked to survival of malignant cells facing ER stress, mediating an adaptive response to chronic UPR signaling^23^.

Despite significant knowledge in other malignancies, the potential function of IRE1α-XBP1s signaling in PCa has not been clear. We recently found that IRE1α activation has a pro-survival role in PCa^11^. The data we presented here show that specific inhibition of IRE1α with MKC8866 has remarkable efficacy to inhibit PCa in mouse models, as well as synergizing with both targeted and chemotherapeutic drugs that are used against PCa in the clinic (Fig. 2). This is significant as patients either do not respond, or eventually develop resistance to PCa therapies that are currently available^10^. To circumvent the development of resistance and to achieve the best response, clinical studies are ongoing to establish combinatorial regimens that have better efficacy than monotherapies^9,24^. In this context, MKC8866 could potentially provide a valuable addition to the spectrum of PCa therapies, especially in tumors that display elevated IRE1α-XBP1s activity upon ADT or chemotherapy.

A previous study has made a connection between the UPR and c-MYC in which PERK-eIF2α-ATF4 signaling was found to mediate the oncogenic effect of c-MYC by activating cytoprotective autophagy in lymphomas^25^. In addition, targeting the kinase activity PERK with a small molecule inhibitor resulted in antitumor and antiangiogenic activity in multiple preclinical models^26^. While these two studies position UPR as a downstream effector of MYC, the current study is the first to show direct activation of MYC expression and its transcriptional output by one of the canonical UPR arms. Taken together, and given the central role of c-MYC in various cancer types, these findings demonstrate that there may be a positive feedback loop between MYC and UPR in cancer, offering opportunities for therapeutic targeting.

**Fig. 6:**
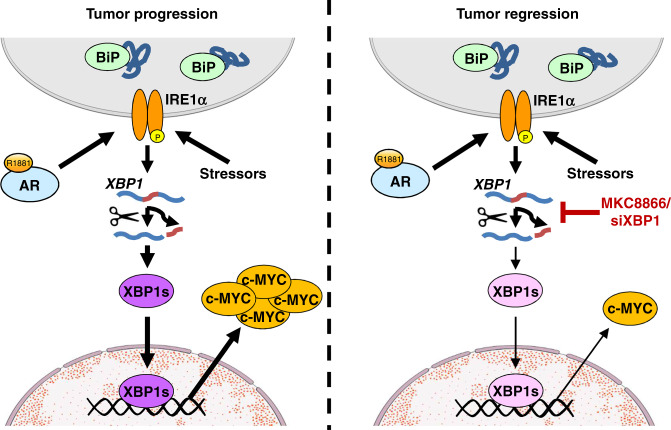
A schematic summary of the present findings. In PCa cells, the androgen receptor (AR) directly activates IRE1α expression, that can also be induced by various stressors on the cell. IRE1α then splices *XBP1* mRNA and allows for XBP1s generation. XBP1s transcriptionally induces c-MYC expression, a potent oncoprotein that contributes to tumor progression (left panel). MKC8866 or siXBP1 inhibit XBP1s expression, resulting in significantly decreased c-MYC levels leading to tumor regression (right panel)

Here we demonstrated, for the first time, that the central oncogenic c-MYC signaling pathway, previously shown to have important roles in PCa progression^13^, is directly activated by IRE1α signaling through XBP1s. Consistently, depleting XBP1s either by the IRE1α RNase-specific inhibitor MKC8866 or siRNA-mediated knockdown results in reduced c-MYC levels and tumor regression (Fig. 6). In addition, XBP1s and c-MYC signaling pathway activation are remarkably correlated in human PCa cohorts (Fig. 4a and Supplementary Fig. 9a). Rescue experiments established that the tumor suppressive activity of MKC8866 is, at least in part, due to inhibition of XBP1s-mediated c-MYC expression (Fig. 4g, h, and Supplementary Fig. 9e and 9f). Despite its important roles in PCa, as well as in other cancer types^12^, previous efforts to inhibit c-MYC function have not been fruitful^27^. The bromodomain inhibitor JQ1 suppresses c-MYC signaling, among its many other effects in PCa, yet our findings indicate that high XBP1s tumors may be more resistant to JQ1 (Supplementary Fig. 9d), due to the potentially higher c-MYC levels. Our findings raise the possibility that c-MYC can be targeted through the IRE1α-XBP1s pathway not only in PCa, but also in other cancer types where c-MYC is important. In addition to c-MYC, our analyses identified other signaling pathways that are also involved in mediating the effects of IRE1α-XBP1s axis in PCa. For example, mTORC1 signaling and fatty acid metabolism-related signaling pathways were significantly affected by MKC8866 treatment or XBP1 knockdown (Fig. 4d); these two are mechanistically important and clinically actionable pathways whose inhibition may synergize with MKC8866. Further work is required to assess this possibility.

During the review process of this manuscript, a study demonstrated that c-MYC is critical for the IRE1α-XBP1s pathway in breast cancer cells by directly activating IRE1α expression and potentiating XBP1s transcriptional activity^28^. Consistently, MKC8866 strongly inhibited c-MYC-driven tumor growth in preclinical models of breast cancer in vivo. Additional work is required to establish if XBP1 activates c-MYC expression in breast cancer cells as well, and whether c-MYC regulates IRE1 expression and XBP1 activity in PCa cells. If so, XBP1s–c-MYC axis will provide a strong feedback loop that establishes a pro-survival pathway in cancer cells that could be targeted at multiple sites for potential therapeutic applications.

PCa is largely indolent: thus, one of the most challenging aspects in the clinic is to identify PCa cases that have an aggressive phenotype compared with the slow-growing, non-dangerous tumors. In this regard, the XBP1s gene signature that we have identified has significant predictive power for disease-free or BCR-free survival in various cohorts (Fig. 5). Additional studies are required to test the utility of this gene signature for disease stratification in the clinic.

In summary, these data demonstrate the pivotal pro-survival role of IRE1α-XBP1s signaling in PCa cells and identify MKC8866 as a candidate drug that could be used in future clinical trials. In addition, our findings establish a direct link between UPR signaling and oncogene activation.

## Methods

### Cell culture

The human PCa cell lines LNCaP and 22Rv1 were purchased from the American Type Culture Collection (LGC Standards); the VCaP cell line was a kind gift of Frank Smit (Radboud University Nijmegen Medical Centre, The Netherlands); the C4-2B cell line was a generous gift of Leland Chung (Cedars-Sinai Medical Center, CA). Cells were routinely kept in a humidified 5% CO_2_ and 95% air incubator at 37 °C in RPMI 1640 containing 10% fetal bovine serum (FBS), 5 mg ml^−1^ penicillin/streptomycin, and 2 mM l-glutamine (Lonza). The LNCaP c-MYC inducible cell line was cultured in RPMI with 10% FBS + puromycin (2 μg ml^−1^) and geneticin (50 μg ml^−1^)^29^. The hormone responsiveness and expression of proteins characteristic to each cell line were tested on a regular basis. For hormone responsiveness experiments, cells were plated in full medium containing 10% FBS and then preincubated in medium containing 5% charcoal-treated (CT)-FBS for 3 days before the induction with the synthetic androgen R1881 (1 nM) for the indicated time periods. All cell lines were routinely tested and were negative of mycoplasma contamination.

### Reagents

XBP1 siRNA was purchased from Santa Cruz (sc-38627), Allstar Negative Control siRNA was from Qiagen (SI03650318), the human pCDNA3-Flag-XBP1s expression plasmid was from GenScript (OHu25513), the pCDNA3-HA-c-MYC was a gift from Martine Roussel (Addgene plasmid # 74164)^30^, and the pGL3-basic and pGL3-MYC luciferase reporter plasmids were kindly provided by Odd Stokke Gabrielsen^31^.

MKC8866 was obtained from Fosun Orinove. Abiraterone acetate (200030) and enzalutamide (201821) were purchased from Medkoo Biosciences. Docetaxel (S1148) and cabazitaxel (S3022) were from Sellekchem and Paclitaxel (P-9600) was from LC Laboratories. JQ1 was a kind gift from Stefan Knapp.

### Cell viability assay

Cells were plated in 96-well plates, treated and grown for 3 days. Cell viability was determined using the Cell Count Kit-8 (CCK-8) (Sigma) according to the manufacturer’s instructions.

### Colony formation assay

Briefly, cells were trypsinized, seeded on plastic dishes (attached) or on top of 0.4% low-melting agarose (unattached), and cultured for the indicated time periods. The cells were then fixed and stained with 0.1% crystal violet, and the total area covered by the colonies was measured using an imaging system (Syngene).

### Prostatosphere assay

Cells (1000 cells ml^−1^) were cultured in suspension in serum-free DMEM-F12 (BioWhittaker) supplemented with B27 (1:50, Invitrogen), 20 ng ml^−1^ EGF (E9644, Sigma), 20 ng ml^−1^ FGF2 (F0291, Sigma), and 4 μg ml^−1^ insulin (I9278, Sigma). For propagation, spheres were collected by gentle centrifugation, dissociated to single cells and then cultured to generate prostatospheres of the next generation. Spheres were imaged after growth for 10 days, and areas covered by spheres was measured and quantified by ImageJ.

### Quantitative PCR

Total RNA was isolated using the TRI Reagent (Sigma-Aldrich, T9424) according to the manufacturer’s instructions and 1 μg of RNA was reverse transcribed using Superscript II (Invitrogen 18064014). The cDNA was used in qPCR whose primer sequences are listed in the Supplementary Information (Supplementary Table 1). A standard curve made from serial dilutions of cDNA was used to calculate the relative amount of the different cDNAs in each sample. The values were normalized to the relative amount of the internal standard GAPDH. The experiments were performed in triplicate and repeated thrice with consistent results.

### Western analysis

Cells were washed once in ice-cold phosphate-buffered saline (PBS) and lysed in radioimmunoprecipitation assay (RIPA) buffer (0.1% SDS, 1% NP-40, 0.5% sodium deoxycholate, 50 mM Tris–HCl pH 8.8, 150 mM NaCl) and cell lysates were boiled at 95 °C for 5 min. Protein samples were then resolved by SDS–PAGE and transferred to a PVDF membrane (Bio-Rad). The blotted membrane was blocked in 5% nonfat dry milk in Tris-buffered saline (TBS) containing 0.1% Tween (TBS-Tween) for 1 h followed by incubation with primary antibody in TBS-Tween containing 5% bovine serum albumin (BSA) for 14–16 h at 4 °C. c-MYC (32072; 1:1000), MAX (199489; 1:1000), and TRRAP (73546; 1:1000) antisera were from Abcam; XBP1s (12782; 1:1000), IRE1α (3294S; 1:1000), phospho-eIF2α (9721L; 1:1000), eIF2α (9722S; 1:1000), BiP (3183; 1:1000), and P58IPK (2940; 1:1000) were from Cell Signaling; AR (sc-816; 1:500), XBP1 (sc-7160; 1:500), ATF6α (sc-166659; 1:500), and GAPDH (sc-47724; 1:2000) were from Santa Cruz; phospho-IRE1α was from Thermo Scientific (PA1-16927; 1:500); TMPRSS2 was from Epitomics (2770-1; 1:1000). The membranes were then incubated with horseradish peroxidase-conjugated anti-rabbit IgG or anti-mouse IgG (Sigma-Aldrich) secondary antibodies in 5% nonfat dry milk dissolved in TBS-Tween for 1 h at room temperature. ECL Western blotting analysis system was utilized for detection of the immunoreactive bands according to the manufacturer’s instructions (Amersham Pharmacia Biotech). Uncropped gels of the representative Western blot results are shown in Supplementary Fig. 10.

### Luciferase reporter assay

LNCaP or 293T cells were cultured in six-well plates, transfected with 1 μg of pGL3-MYC luciferase reporter plasmid plus either empty vector (pCDNA3) or the pCDNA3-Flag-XBP1s plasmid of the indicated concentration for 24 h before harvest. Luciferase activity was determined using a luciferase assay system (Promega) and a Wallac Victor^2^ 1420 Multilabel counter (Perkin Elmer).

### Chromatin immunoprecipitation

ChIP experiments were carried out according to the standard protocol (Upstate Biotechnology) with some modifications. Briefly, cells were treated as indicated before cross-linking with 1% formaldehyde at 37 °C. The cells were then quenched with 125 mM glycine. Chromatin was sheared using the Bioruptor sonicator (Diagenode). After centrifugation, sheared chromatin was immunoprecipitated overnight with a Flag antibody (A00187, GenScript), or mouse IgG. Antibody bound chromatin complexes were then immunoprecipitated with protein A-agarose beads, and eluted in SDS buffer. Formaldehyde cross-linking was reversed at 65 °C overnight, followed by DNA purification. Immunoprecipitated DNA, as well as input DNA, was quantified by qPCR using specific primer sets as indicated. Primer sequences are available upon request.

### Xenografts

All procedures on mice, including mouse strain, sex, age, number of animals allowed, and housing, were conducted according to an experimental protocol approved by the University of Oslo Institutional Animal Care and Use Committee. Briefly, cells were grown in vitro and for each injection suspended in 50 μl RPMI-1640 medium and mixed with 50 μl matrigel (BD Biosciences). The mixture was then subcutaneously inoculated into male nude mice (BALB/c Nu/Nu, 5 weeks of age) in both hind flanks.

After palpable tumors appeared, the mice were randomly grouped and daily received either 300 mg kg^−1^ MKC8866 or vehicle (1% microcrystalline in 1 g ml^−1^ sucrose). For the combinatorial experiment of MKC8866 and enzalutamide, mice were assigned into four groups (*n* = 6 per group): oral gavage of 300 mg kg^−1^ MKC8866 every other day, oral gavage of 30 mg kg^−1^ enzalutamide every other day, a combination of daily gavage of either MKC8866 or enzalutamide, and daily gavage of either MKC8866 or 0.5% HP methyl cellulose with 0.1% Tween 20 as a vehicle control. The four groups (*n* = 6 per group) for the combination of MKC8866 and abiraterone acetate were: oral gavage of 300 mg kg^−1^ MKC8866 every other day, oral gavage of 20 mg kg^−1^ abiraterone acetate every other day, a combination of two treatments, and corresponding vehicles. The four groups (*n* = 6 per group) for the combination of MKC8866 and cabazitaxel were: oral gavage of 300 mg kg^−1^ MKC8866 every other day, intraperitoneal injections of 5 mg kg^−1^ cabazitaxel twice a week, a combination of two treatments, and corresponding vehicles. Tumor weight was measured in the end of experiment.

For assessment of potential synergistic effects: assuming the fractional response to drug A alone is Fa, and similarly that of drug B alone is Fb, synergy is defined when the combinatorial fractional response Fc > Fa*(1 − Fb). For a review see ref. ^32^.

### Histological and immunohistochemical staining

After de-paraffinization, the tumor sections were first stained with hematoxylin and then eosin following the manufacturer’s guidelines (Thermo Fisher Scientific) for histological evaluation. Necrotic content was scored and quantified using ImageJ. For immunohistochemistry, antigen retrieval was done by autoclaving at 120 °C for 10 min in 10 mM citrate buffer (pH 6.4) following de-paraffinization. The primary antibodies PCNA (P8825, Sigma; 1:200), cleaved caspase-3 (#9664, Cell Signaling; 1:200), c-MYC (32072, Abcam; 1:100), and XBP1s (a kind gift from Eric Chevet; 1:200)^33^ were used for overnight incubation at 4 °C. The Supersensitive Detection kit (Biogenex) was used for antigen detection.

Primary prostate tumors were prospectively collected at the Norwegian Radium hospital, University of Oslo, and consecutive tumor sections were generated for immunohistochemical analysis of XBP1s and c-MYC colocalization. The study was approved by the South-East Norway Health Authority Regional Ethics Committee.

Human PCa specimens (*n* = 260) were obtained from Vancouver Prostate Centre Tissue Bank. The H&E slides were reviewed, and the desired areas were used to construct TMAs (Beecher Instruments, MD, USA). All specimens were from radical prostatectomies. IHC was conducted by Ventana auto-stainer model Discover XT (Ventana Medical System, Tuscan, Arizona) with enzyme-labeled biotin streptavidin system and solvent-resistant DAB Map kit. For scoring, values on a four-point scale were assigned to each immunostain. Descriptively, 1 represents no apparent staining or very weak level of staining, 2 represents a faint or focal, questionably present stain, 3 represents a stain of convincing intensity in a minority of cells, and 4 represents a stain of convincing intensity in a majority of cells. SPSS 10.0 software was used for IHC statistical analysis.

### RNA sequencing

For siXBP1 group, LNCaP cells were reverse-transfected with either Allstar control siRNA or XBP1 siRNA, after 2 days the cells were treated with 30 nM TG for 3 h; for MKC8866 group, LNCaP cells were treated with either DMSO or 10 μM MKC8866 for 1 day before being induced with 30 nM TG for 3 h. After RNA extraction, the sequencing was performed at the Norwegian Sequencing Center, where the TruSeq stranded RNA sample prep kit was used for library generation, and 150 bp paired end sequencing was performed using an Illumina HiSeq 3000 instrument (Illumina, Inc.). All sequence data sets have been submitted to GEO (GSE103864).

### Gene expression quantification

Raw sequencing reads were aligned onto the human reference genome (hg38) using the STAR software (version 2.5.2)^34^. Read summarization was performed using the featureCounts function of the Subread package^35^ and the gene annotation file *Homo_sapiens.GRCh38.85.gtf* downloaded from the ENSEMBL annotation server.

### XBP1 isoform expression quantification

Isoform-specific XBP1 expression was quantified using the splice-loci output generated from the STAR alignments. The relative abundance of the spliced XBP1 isoform was estimated as the number of spliced reads that mapped from chr22: 28,796,122 to chr22: 28,796,147 (hg38) per million total spliced reads. Differential expression of isoform levels was evaluated using a negative binomial generalized linear model as implemented in the *glm.nb* function of the MASS R package. To account for differences in library depth, the total number of reads sequenced was added as an additional covariate into the model.

### Differential expression analysis

Differential expression analysis was conducted using the R statistical software in conjunction with the DESeq2 package^36^. In short, samples were normalized for sequencing depth using the standard DESeq2 function *estimateSizeFactors*. The DESeq2 modeling framework is based on the negative binomial distribution and uses the DESeq2 normalized gene counts as input. Two independent models were used to test differential expression between the siXBP1 and siCtrl and between the MKC8866 and vehicle groups. Analysis was restricted to genes with more than five counts in at least one sample per group.

### Overlap analysis

To assess the statistical significance of overlap between the top 100 differentially expressed genes in the two treatments, a binomial test was used. The hypothesized probability of success was estimated as 100 divided by the number of genes included in the differential expression analysis.

### Hallmark pathway enrichment analysis

Hallmark pathway enrichment analysis was performed to help the interpretation of the set of up-regulated or down-regulated genes derived from the differential expression analyses. The hallmark gene set annotation was downloaded from the Molecular Signature Database v6.0 (http://software.broadinstitute.org/gsea/msigdb/index.jsp). This annotation contains 50 gene sets and each gene set was independently tested for enrichment. To statistically assess the enrichment, we performed a Kolmogorov–Smirnov test comparing the distribution of log2 fold changes between genes annotated to a given gene set and all other genes.

### Bioinformatics analysis

The concordance of AR activity^37^ and expression of IRE1-regulated genes^38^ in PCa specimens is evaluated using the published datasets^39–42^. The samples were stratified into two groups based on AR activity levels, and expressions of prominent IRE1-regulated genes were compared. The statistical significance was determined by *t*-test.

The transcriptional activity of XBP1s was scored using a gene set including 36 XBP1s specific targets generated from this study and a previous report^38^. The transcriptional activity of c-MYC was predicted using the SCHUHMACHER_MYC_TARGETS_UP^43^. The correlation between XBP1s signaling and c-MYC signaling was evaluated using published PCa gene expression datasets, including TCGA cohort^41^, MSKCC cohort^40^, SU2C cohort^39^, GSE94767^42^, GSE54460^44^, GSE70768^45^, GSE6919^46,47^, GSE16560^48^, GSE35988^49^, GSE77930^50^, and GSE7076^51^.

RNA-seq data were analyzed using GSEA software to identify functionally related groups of genes (gene sets) with statistically significant enrichment^52,53^. The XBP1s-regulated genes identified here were subjected to differential expression analysis in normal and cancer tissues of prostate from multiple published PCa datasets^39–42^. Genes that showed significant increase in tumor compared with benign prostate were used in survival analysis with multiple PCa cohorts. Genes whose expression associated with poor prognosis were selected for further analysis.

For identification of potential XBP1s sites in the *c-MYC* flanking regions, we have used the transcription-binding site prediction PROMO program (http://alggen.lsi.upc.edu/); this was done using the default setting of 15% maximum matrix dissimilarity rate that is stringent. PROMO uses XBP-1-binding sites (CTCGAGATG) defined in the TRANSFAC database (version 8.3) (http://genexplain.com/transfac/) to construct weight matrices for the prediction. Under the given settings, we identified three binding regions within the overlapping parts of the two primer pairs used in the ChIP-qPCR experiment.

### Statistical analysis

Data are presented as mean and standard error of mean. All values were calculated using Microsoft Excel software. The treatment effects in each experiment were compared by two-sided *t*-test. Differences between groups were considered significant at *P* < 0.05.

### Supplementary information

Supplementary Information

Description of Additional Supplementary Files

Supplementary Data 1

## Data Availability

All relevant data are available within the manuscript and its supplementary information, or from the authors upon request. Please contact Yang Jin (yang.jin@ibv.uio.no) or Fahri Saatcioglu (fahris@ibv.uio.no) for any requests. All sequence data sets have been submitted to GEO (GSE103864). A reporting summary for this article is available as a Supplementary Information file.
