# Author Correction: IRE1α-XBP1s pathway promotes prostate cancer by activating c-MYC signaling

**DOI:** 10.1038/s41467-024-50645-x

**Published:** 2024-07-23

**Authors:** Xia Sheng, Hatice Zeynep Nenseth, Su Qu, Omer F. Kuzu, Turid Frahnow, Lukas Simon, Stephanie Greene, Qingping Zeng, Ladan Fazli, Paul S. Rennie, Ian G. Mills, Håvard Danielsen, Fabian Theis, John B. Patterson, Yang Jin, Fahri Saatcioglu

**Affiliations:** 1 Department of Biosciences, University of Oslo, 0316 Oslo, Norway; 2 School of Public Health, Tongji Medical College, Huazhong University of Science and Technology, 430030 Wuhan, China; 3 Institute of Computational Biology, Helmholtz Zentrum München, 85764 Neuherberg, Germany; 4 Faculty of Business Administration and Economics, Chair DataScience, University Bielefeld, 33615 Bielefeld, Germany; 5 Fosun Orinove, Inc., Unit 211, Building A4, 218 Xinhu Street, 215000 SuZhou, China; 6 The Vancouver Prostate Centre, Vancouver, BC V6H3Z6 Canada; 7 Movember/PCUK Centre of Excellence for Prostate Cancer Research, Centre for Cancer Research and Cell Biology (CCRCB), Queen’s University of Belfast, Belfast, BT7 1NN UK; 8 Institute for Cancer Genetics and Informatics, Oslo University Hospital, 0379 Oslo, Norway; 9 Center for Cancer Biomedicine, University of Oslo, 0316 Oslo, Norway; 10 Department of Informatics, University of Oslo, 0316 Oslo, Norway; 11 Nuffield Division of Clinical Laboratory Sciences, University of Oxford, Oxford, OX3 7LF UK

Correction to: *Nature Communications* 10.1038/s41467-018-08152-3, published online 24 January 2019

The original version of the manuscript contained errors in Figure 4 and Supplementary Figs. 1a and 2d.

In detail:

- In Figure 4h, the sphere images “PS+Dox” and PS-Dox” were inadvertently duplicated.
- In Figure 4e, the label for the second row of blots should read “XBP1” instead of “XBP1s”
- In Supplementary Fig. 1a, the duplicated first and last bands of the IRE1α blots were due to an incorrect selection of lanes from the original full blots. In the same figure, the correct IRE1α bands of the C4-2B samples are now also shown.
- In Supplementary Fig. 2d, the incorrect western blot image for IRE1α and GAPDH in 22Rv1 cells were presented.

The updated Figure 4 is shown below and the updated Supplementary Information file can be found online associated with this correction.


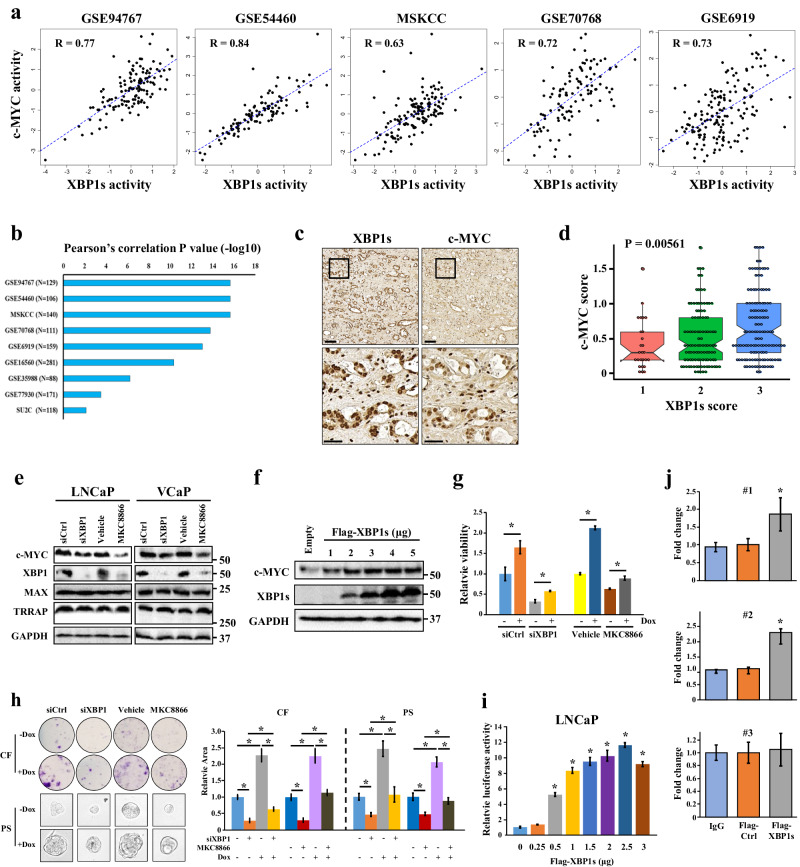


The raw data of Figure 4h and the full blots of Supplementary Fig. 1a and Supplementary Fig. 2d are also available at Figshare: https://figshare.com/articles/figure/Raw_data_for_Sheng_et_al_2019_Erratum_zip/25790655.

Please also note that for all western blots, in order to analyze multiple proteins, the same samples were run on multiple gels and blotted with different antibodies. This information should have been included in the “Methods” section.

### Supplementary information

Updated Supplementary Information

