# Supplementary material for: IRE1α-XBP1s pathway promotes prostate cancer by activating c-MYC signaling
Source: Nat Commun. 2019 Jan 24;10:323. doi: 10.1038/s41467-018-08152-3 (PMC6345973; doi:10.1038/s41467-018-08152-3)
Supplement: Supplementary file 2 — Description of Additional Supplementary Files [file 41467_2018_8152_MOESM2_ESM.docx]

**Description of Additional Supplementary Files**

File Name: Supplementary Data 1

Description: List of 733 genes significantly downregulated by siXBP1 or MKC8866 in the RNA-seq analysis.
