# Supplementary material for: Author Correction: IRE1α-XBP1s pathway promotes prostate cancer by activating c-MYC signaling
Source: Nat Commun. 2024 Jul 23;15:6190. doi: 10.1038/s41467-024-50645-x (PMC11266509; doi:10.1038/s41467-024-50645-x)
Supplement: Supplementary file 1 — Updated Supplementary Information [file 41467_2024_50645_MOESM1_ESM.pdf]

Supplementary Information

IRE1α-XBP1s pathway promotes prostate cancer by activating c-MYC signaling

Sheng *et al*

Supplementary Figures

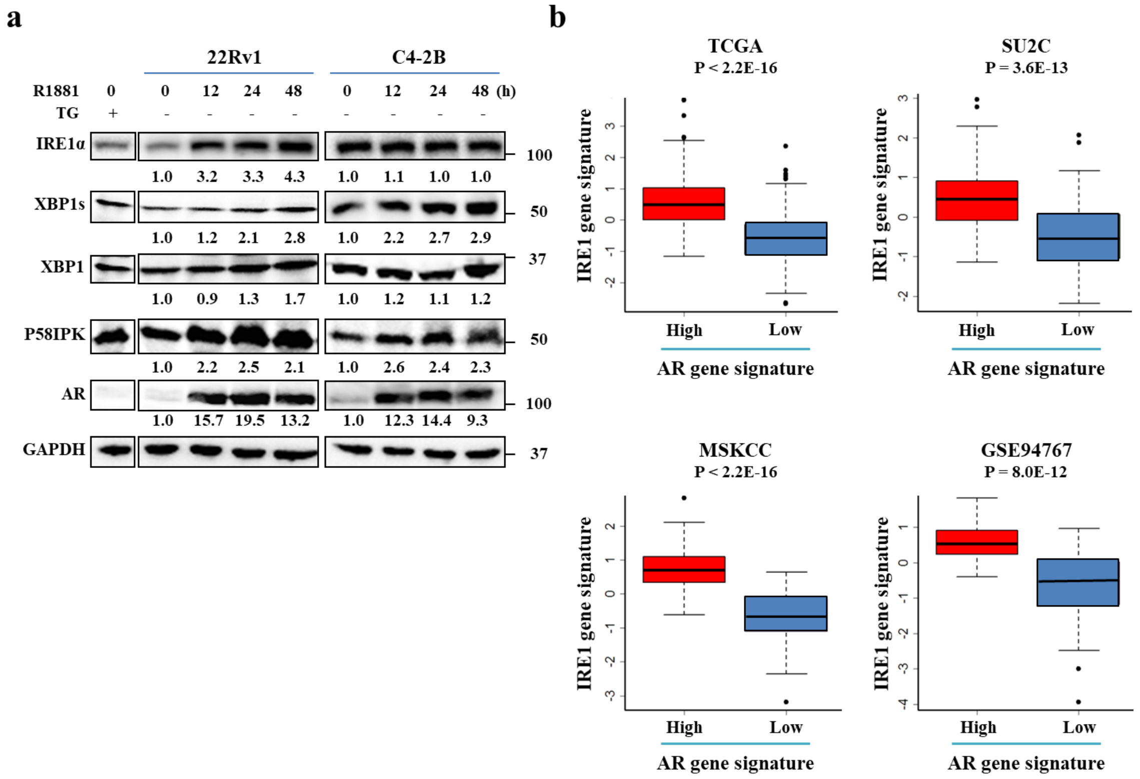

Supplementary Figure 1. Androgen signaling is activated in CRPC models and patient samples. **(a)** 22Rv1 and C4-2B cells were cultured under hormone deprived conditions for 72 h and then treated with 1 nM R1881 for the indicated times. Protein expression was determined by Western analysis. Extracts from 22Rv1 cells treated with 30 nM thapsigargin (TG) for 3 h were used as a positive control. To analyze multiple proteins, the same samples were run on multiple gels and blotted with different antibodies. Quantification of IRE1α and XBP1s normalized to GAPDH expression was indicated below each corresponding blot. **(b)** Gene expression data from four different human PCa patient cohorts were grouped into high and low AR expression and level of IRE1 arm gene expression was compared between the two groups. Thick horizontal lines represent the median, and the boxes represent the upper and lower quartiles. The whiskers represent the 5th and 95th percentiles, and the outliers are presented as dots. P values for the different genes are indicated, student t-test.

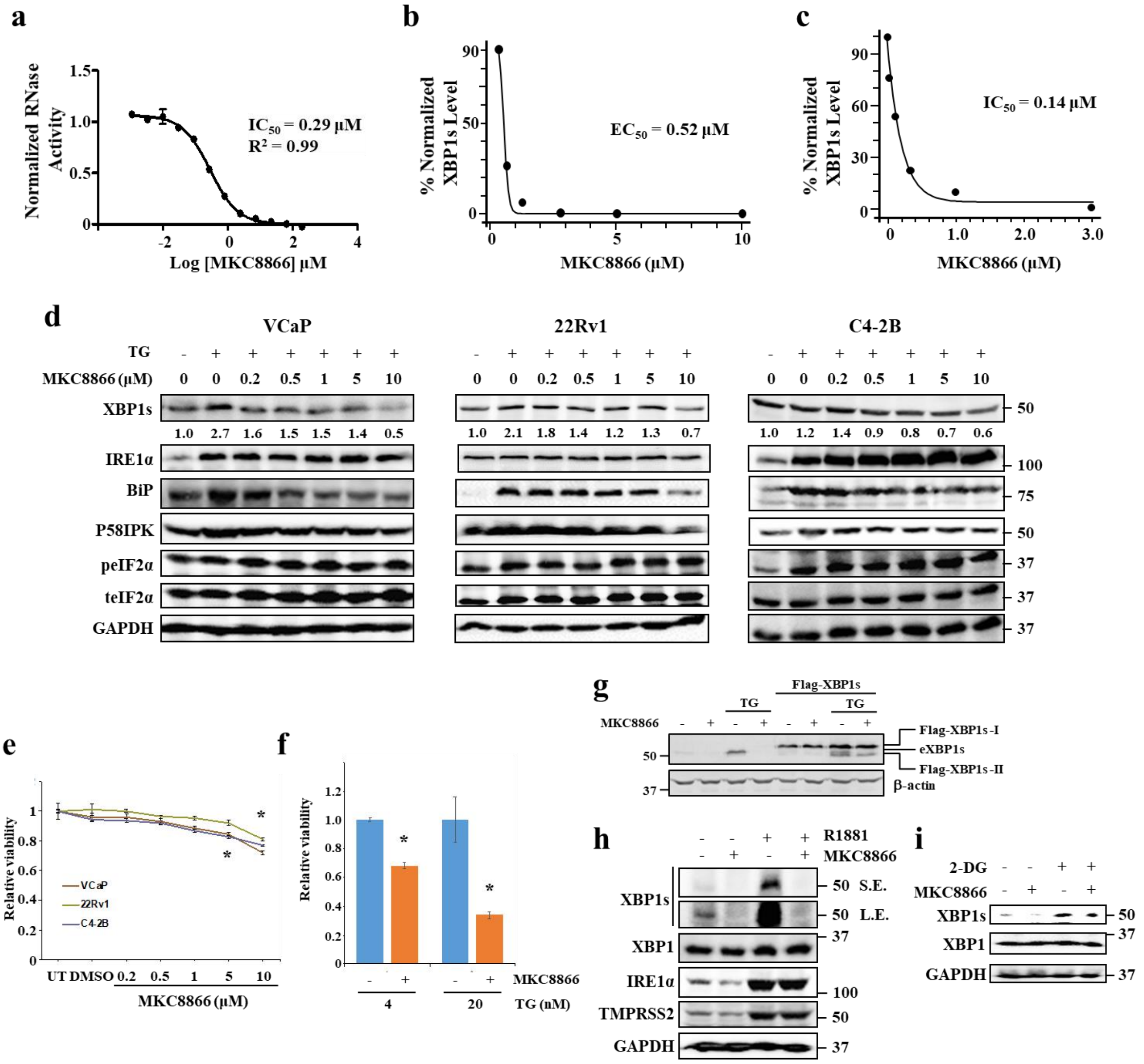

Supplementary Figure 2. Pharmacokinetic properties of MKC8866. **(a)** MKC8866 was pre-incubated with 10 nM recombinant IRE1α for 1 h, 100 nM single hairpin RNA substrate was added, and cleavage activity was recorded. Data shown are at 30 min after substrate addition. **(b)** MM1 cells were treated with 1 mM DTT together with MKC8866 and *XBP1s* levels were determined by qPCR. **(c)** Unstressed RPMI 8226 cells were treated with increasing amounts of MKC8866 and *XBP1s* level was determined by qPCR. **(d)** VCaP, 22Rv1 and C4-2B cells were cultured in regular growth medium and treated with 30 nM TG and the indicated doses of MKC8866 for 24 h. Protein expression was then determined by Western analysis. To analyze multiple proteins, the same samples were run on multiple gels and blotted with different antibodies. **(e)** VCaP, 22Rv1 and C4-2B cells were either left untreated (UT), or treated with

vehicle (DMSO) or MKC8866 with indicated doses. Cell viability was measured after 3 days. **(f)** C4-2B cells were treated with either DMSO or 10 μM MKC8866 in the presence of 4 nM or 20 nM TG. Cell viability was measured after 48 h. **(g)** LNCaP cells were cultured in 5% CT-FBS medium, transfected with 3 μg of either empty vector or flag-XBP1s, treated with or without 10 μM MKC8866 for 24 h. Prior to cell harvest and western analyses, the cells were either left untreated or treated with 30 nM TG for 5 h as indicated. Primary Flag-XBP1s band (-I), the TG-induced minor Flag-XBP1 species (-II), and the endogenous-XBP1s (e-XBP1s) band are indicated. Beta-actin was used as loading control (bottom). Molecular weight markers are shown on the left. **(h)** LNCaP cells were hormone starved in 5% CT-FBS media for 2 days before treatment with either ethanol or 1 nM R1881, in addition to DMSO as control or 10 μM MKC8866. Cells were harvested after 24 h for Western analysis. Both short exposure (S.E.) and long exposure (L.E.) blots for XBP1s are shown. **(i)** LNCaP cells were treated with either ethanol or 2 mM 2-deoxyglucose (2-DG), plus either DMSO or 10 μM MKC8866. Cells were harvested after 24 h for Western analysis. *$P<0.05$, student t-test, error bars denote the SD.

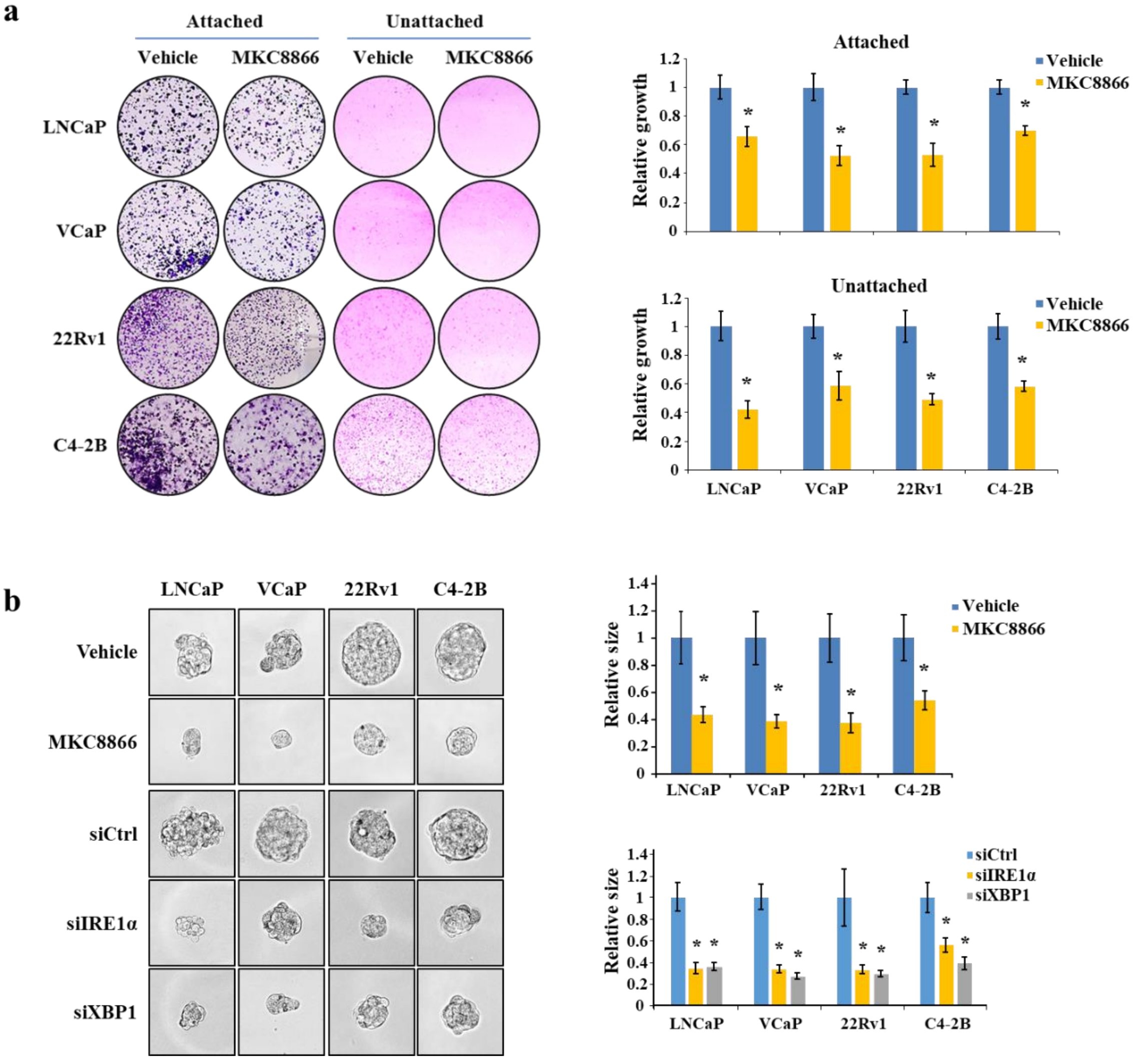

Supplementary Figure 3. MKC8866 inhibits formation of colonies and prostatospheres of various PCa cell lines in vitro. **(a)** LNCaP, VCaP, 22Rv1 and C4-2B cells were cultured in regular growth medium on plastic dishes (attached) supplemented with either DMSO or 10 µM MKC8866, or cultured in regular growth medium containing 0.2% agarose on top of 0.4% agarose (unattached). Colonies were visualized and quantified after 1 or 2 weeks. **(b)** LNCaP, VCaP, 22Rv1 and C4-2B cells were seeded in low-attachment plates under the condition that allows for prostatospheres, in the presence of either DMSO or 10 µM MKC8866, alternatively transfected with siRNAs of control, IRE1α or XBP1. The spheres were imaged, the sizes were measured and quantified. *P<0.05, student t-test, error bars denote the SD.

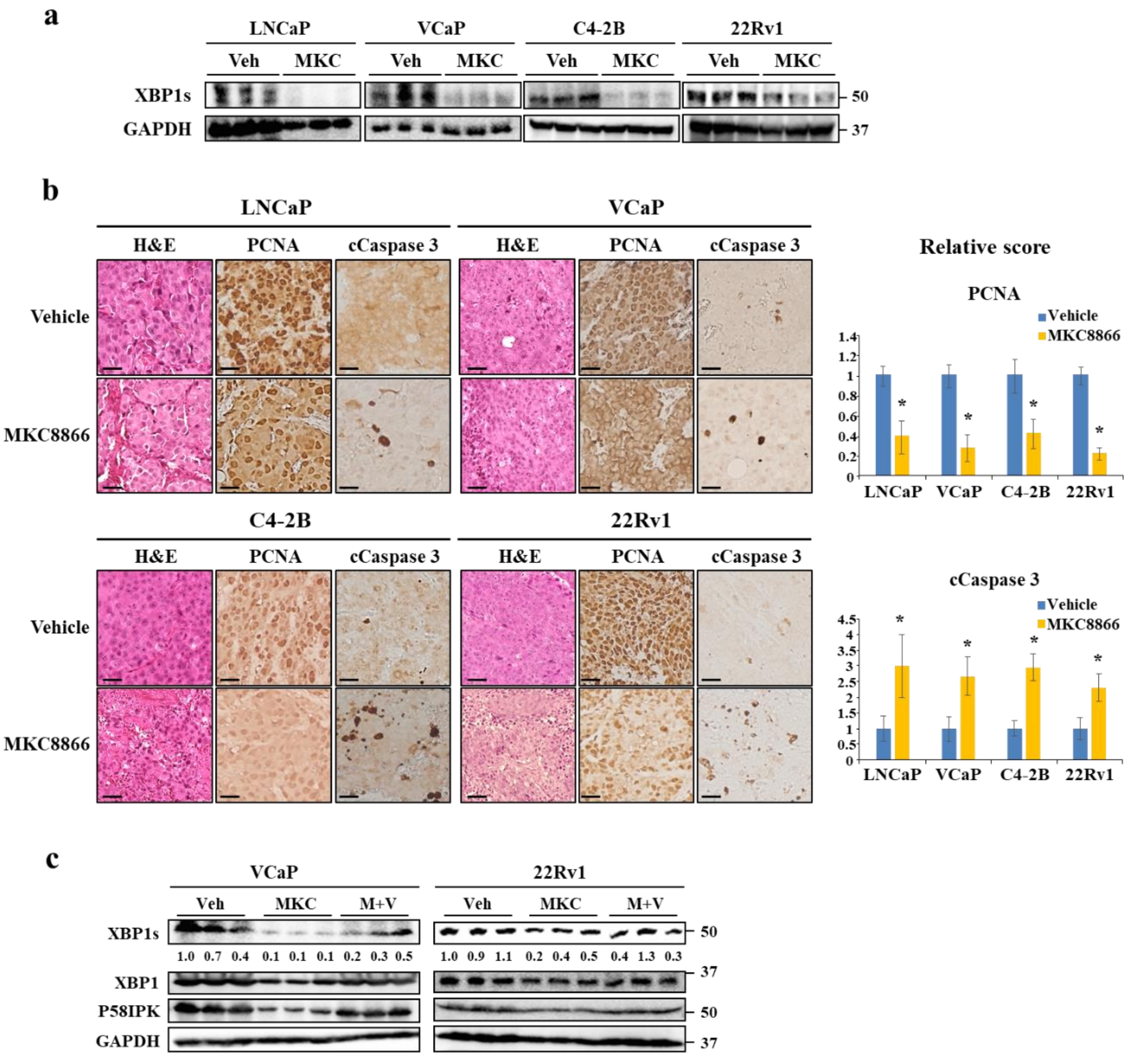

Supplementary Figure 4. Analyses of the in vivo effect of MKC8866 in multiple PCa xenograft models. **(a)** Western analyses of XBP1s levels in tumors presented in Fig. 2a. **(b)** Sections of tumors presented in Fig. 2a were stained with either hematoxylin and eosin (H&E), or immunostained for PCNA and cleaved Caspase-3 (cCaspase-3). The staining was then scored and quantified. Scale bars, 25 μM. *P<0.05, student t-test, error bars denote the SD. **(c)** Western analyses of XBP1s, XBP1 and P58IPK levels in tumors presented in Fig. 2b.

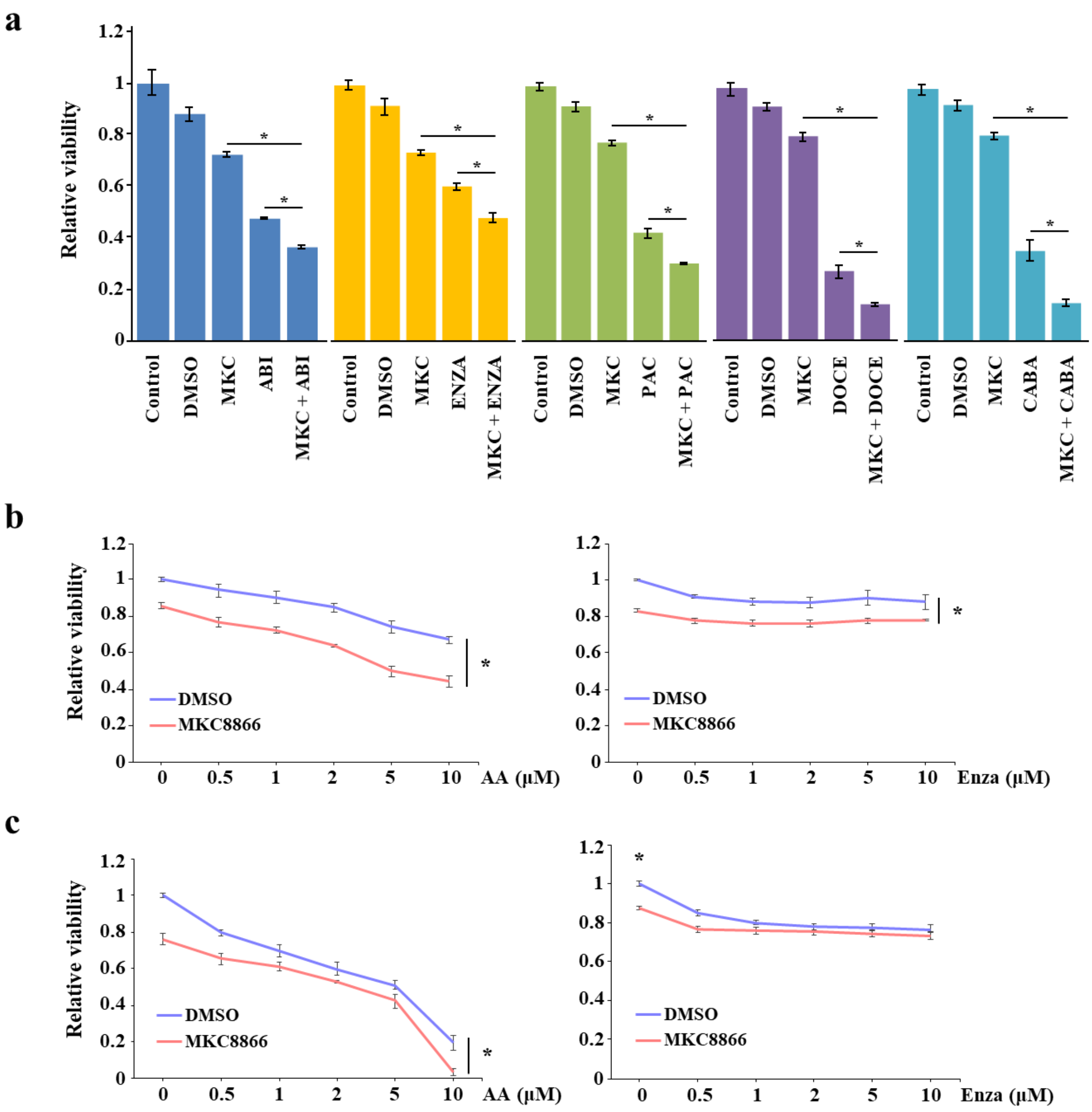

Supplementary Figure 5. Combinatorial effects of MKC8866 with PCa drugs used in the clinic *in vitro*. **(a)** LNCaP cells were seeded in 96-well plates and left either untreated (UT), treated with DMSO, MKC8866 (MKC, 5 μM) or a clinical drug alone, or a combination of both. Cell viability was measured using the CCK8 assay after 3 days. The clinical drugs include abiraterone acetate (ABI, 5 μM), enzalutamide (ENZA, 5 μM), paclitaxel (PAC, 5 nM), docetaxel (DOCE, 10 nM), and cabazitaxel (CABA, 2 nM). **(b)** 22Rv1 cells were seeded in 96-well plates, treated with either DMSO or 10 μM MKC8866, combined with abiraterone acetate (AA) or enzalutamide (Enza) with increasing concentrations as indicated. Cell viability was

measured after 3 days. **(c)** Similar experiment as in **b** was performed using C4-2B cells. *P<0.05, student t-test, error bars denote the SD.

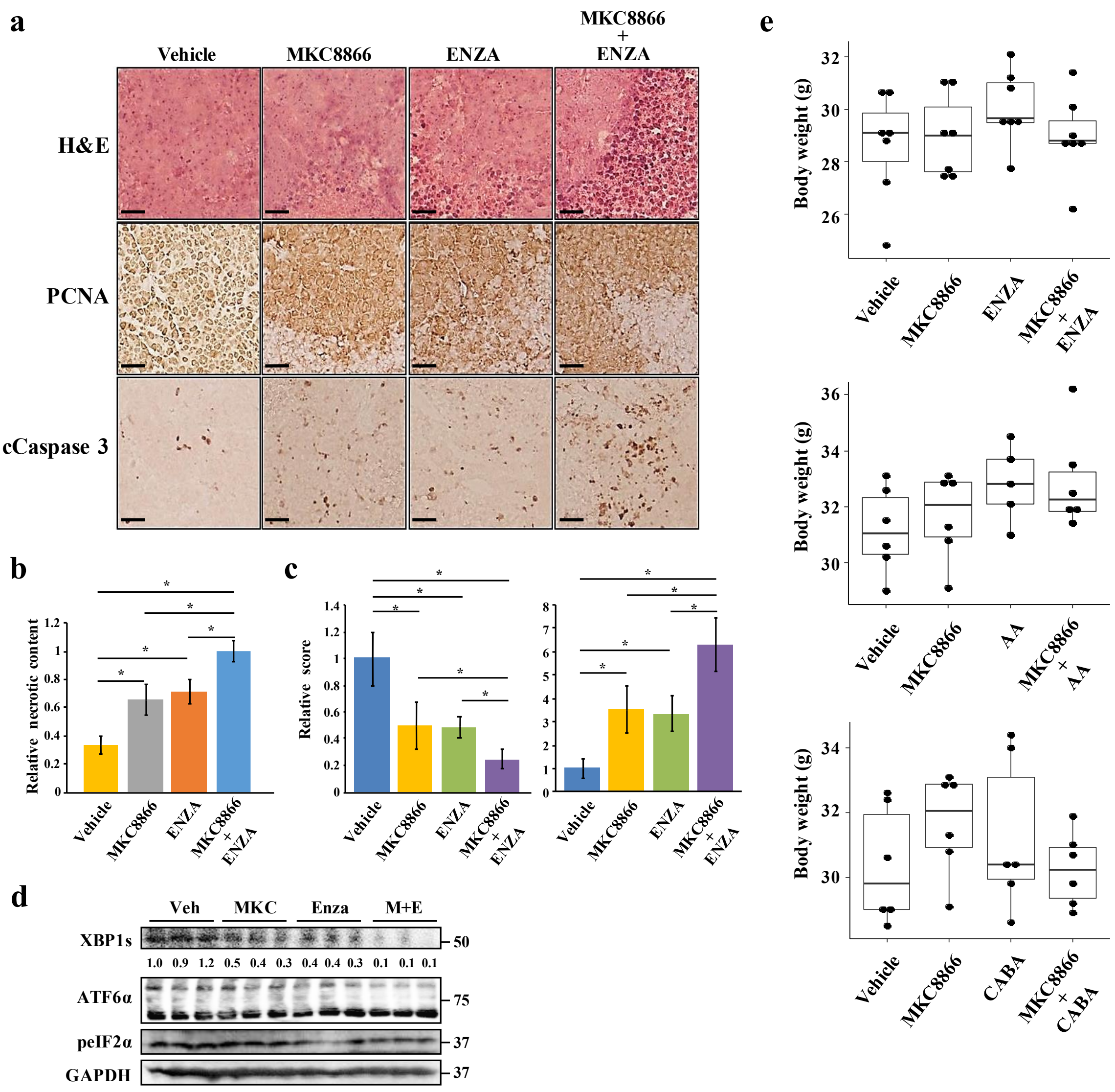

Supplementary Figure 6. Combinatorial effects of MKC8866 with clinical PCa drugs *in vivo*. **(a)** H&E and immunohistochemical stainings of tumors from the experiment described in Fig. 2d. Scale bars, 40 μM. **(b)** Necrotic area of three different tumor sections in each group was scored and quantified. P values were calculated by Student's t-test. **(c)** PCNA and cleaved caspase 3 staining was scored and quantified. P values were calculated by the Student's t-test. **(d)** Western analyses of tumors harvested in Fig. 2d. Veh, vehicle; MKC, MKC8866; Enza, enzalutamide; M+E, MKC8866 plus enzalutamide. **(e)** Body weights of mice in Fig. 2d-f were measured and compared at the end of the experiment. *P<0.05, student t-test, error bars denote the SD.

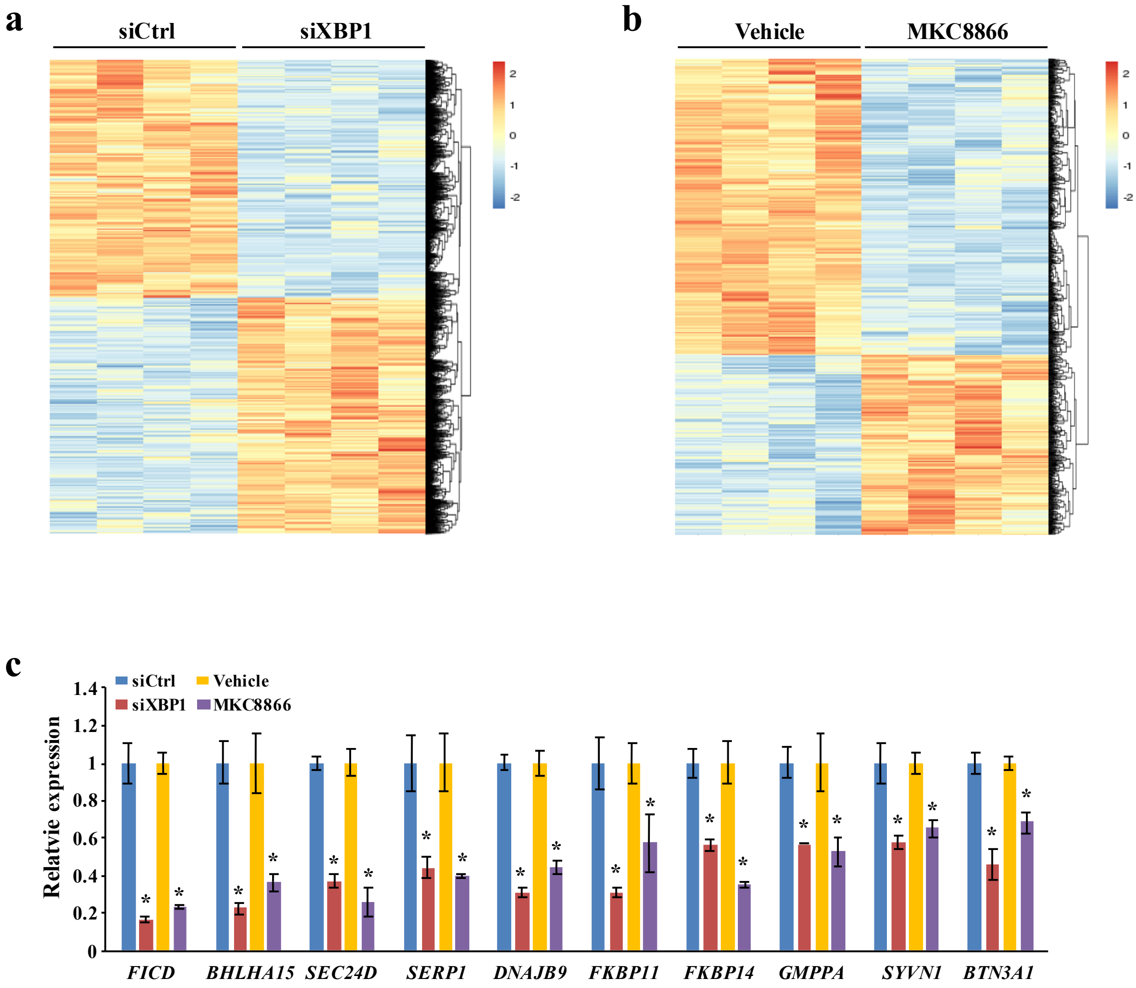

Supplementary Figure 7. Presentation and validation of the RNA-seq analyses. **(a)** Expression of top 100 genes identified in the RNA-seq analysis in XBP1 knockdown cells as differentially regulated are represented in a heat map. Each column represents an independent sample (quadruple). **(b)** Same as in **a**, but the analysis was done on the MKC8866-treated samples. **(c)** 10 of the top downregulated genes were validated by qPCR. Data are from an experiment done in triplicate. *P<0.05, student t-test, error bars denote the SD.

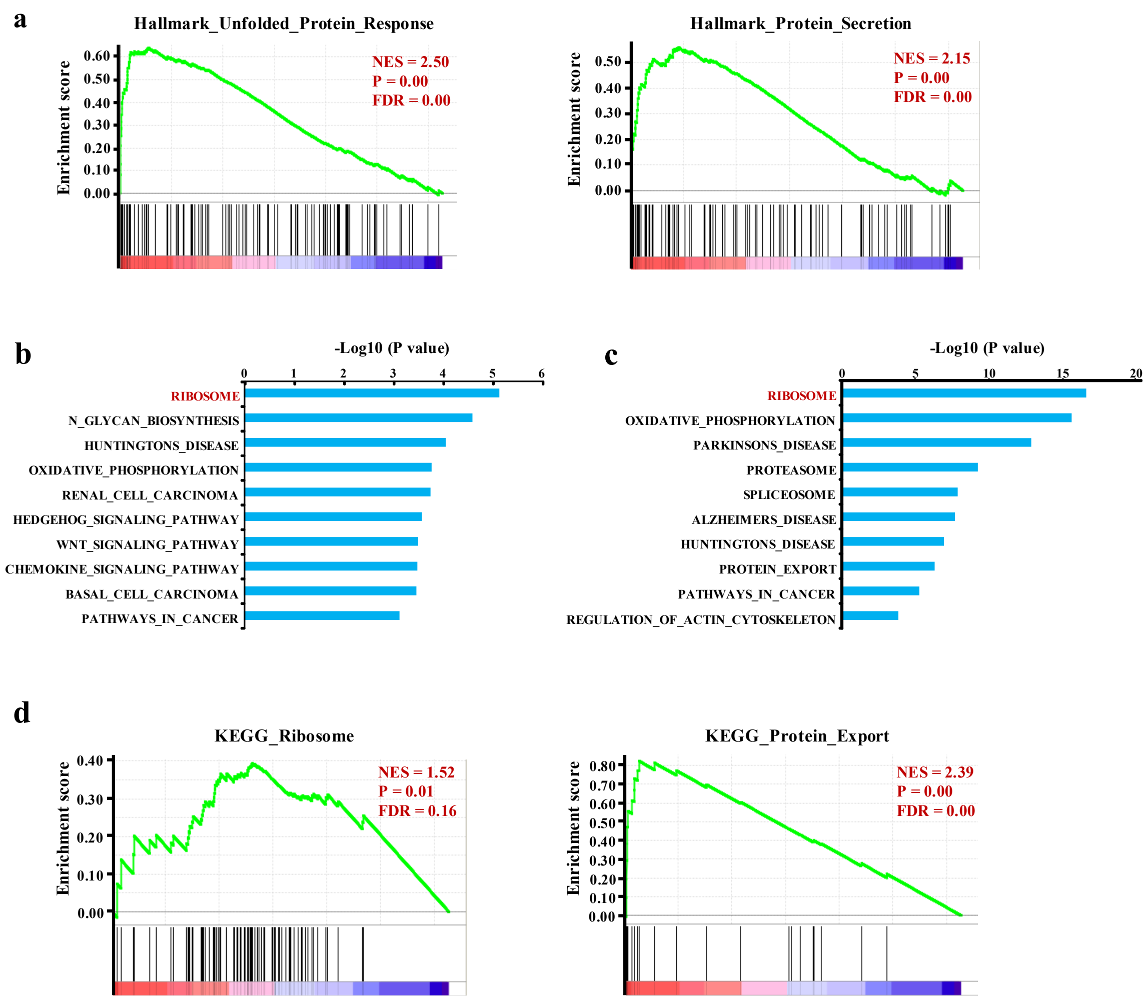

Supplementary Figure 8. GSEA and KEGG analyses of the RNA-seq data. **(a)** GSEA revealed positive enrichment of XBP1 target genes in Hallmark pathways Unfolded Protein Response and Protein Secretion. **(b)** KEGG analysis of siXBP1 RNA-seq data. **(c)** KEGG analysis of MKC8866 RNA-seq data. **(d)** GSEA revealed positive enrichment of XBP1 target genes in KEGG Ribosome and Protein Export.

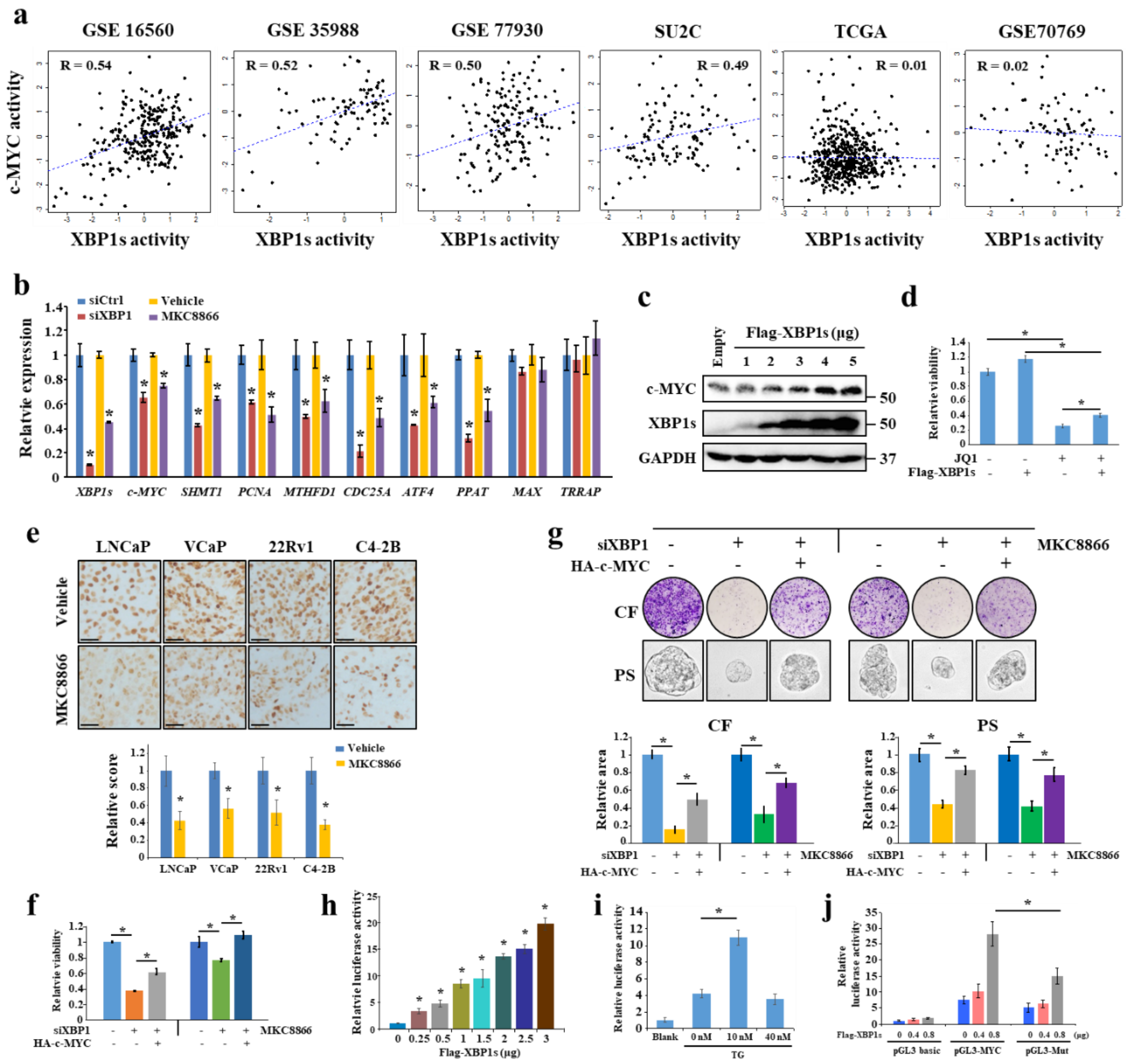

Supplementary Figure 9. Direct regulation of C-MYC expression by XBP1s in prostate cancer. **(a)** The scatter plots show the relationship between target genes expression of XBP1s and c-MYC in six independent PCa datasets. **(b)** mRNA expression of *c-MYC*, its representative targets *SHMT1*, *PCNA*, *MTHFD1*, *CDC25A*, *ATF4* and *PPAT*, as well as *MAX* and *TRRAP* in XBP1 knockdown or MKC8866-treated LNCaP cells. **(c)** VCaP cells were transfected with either empty vector or flag-XBP1s expression vector. XBP1s and c-MYC levels were then determined by Western analysis after 2 days. **(d)** LNCaP cells were seeded in 96-well plates and transfected with either empty vector (pCDNA3) or the pCDNA3-Flag-XBP1s plasmid. Cells were then treated with either vehicle or 5 μM JQ1. Cell viability was measured after 3 days. **(e)** Sections from xenograft tumors described in Fig. 2a were immunostained and scored

for c-MYC expression. Representative images are shown. Scale bars, 40 μM. **(f)** LNCaP cells were either treated with siXBP1 or MKC8866, transfected with an expression vector for HA-tagged c-MYC, and grown in 10% CT-FBS medium. Cell viability was measured 3 days after HA-c-MYC transfection. **(g)** Same procedure was performed as in **e**, colony formation (CF) was measured after two weeks while prostatospheres (PS) after one week. **(h)** 293T cells were cultured in 5% CT-FBS medium, transfected with 1 μg of pGL3-MYC luciferase reporter plasmid plus either empty vector (pCDNA3) or the pCDNA3-Flag-XBP1s plasmid. Luciferase activity was determined after 24h. Results are from a representative experiment in triplicate. **(i)** 293T cells were cultured in 5% CT-FBS medium, transfected with 1 μg of pGL3-MYC luciferase reporter plasmid and treated with TG of the indicated dose for 6 h. Luciferase activity was determined. Results are from a representative experiment performed in triplicate. **(j)** 293T cells were cultured in 5% CT-FBS medium, transfected with 1 μg of pGL3 basic, pGL3-MYC or pGL3-MYC deletion mutant (pGL3-Mut) luciferase reporter plasmid plus either empty vector (pCDNA3) or the pCDNA3-Flag-XBP1s plasmid. Luciferase activity was determined after 24h. Results are from a representative experiment in triplicate. *$P<0.05$, student t-test, error bars denote the SD.

Uncropped Western blots

Figure 1b

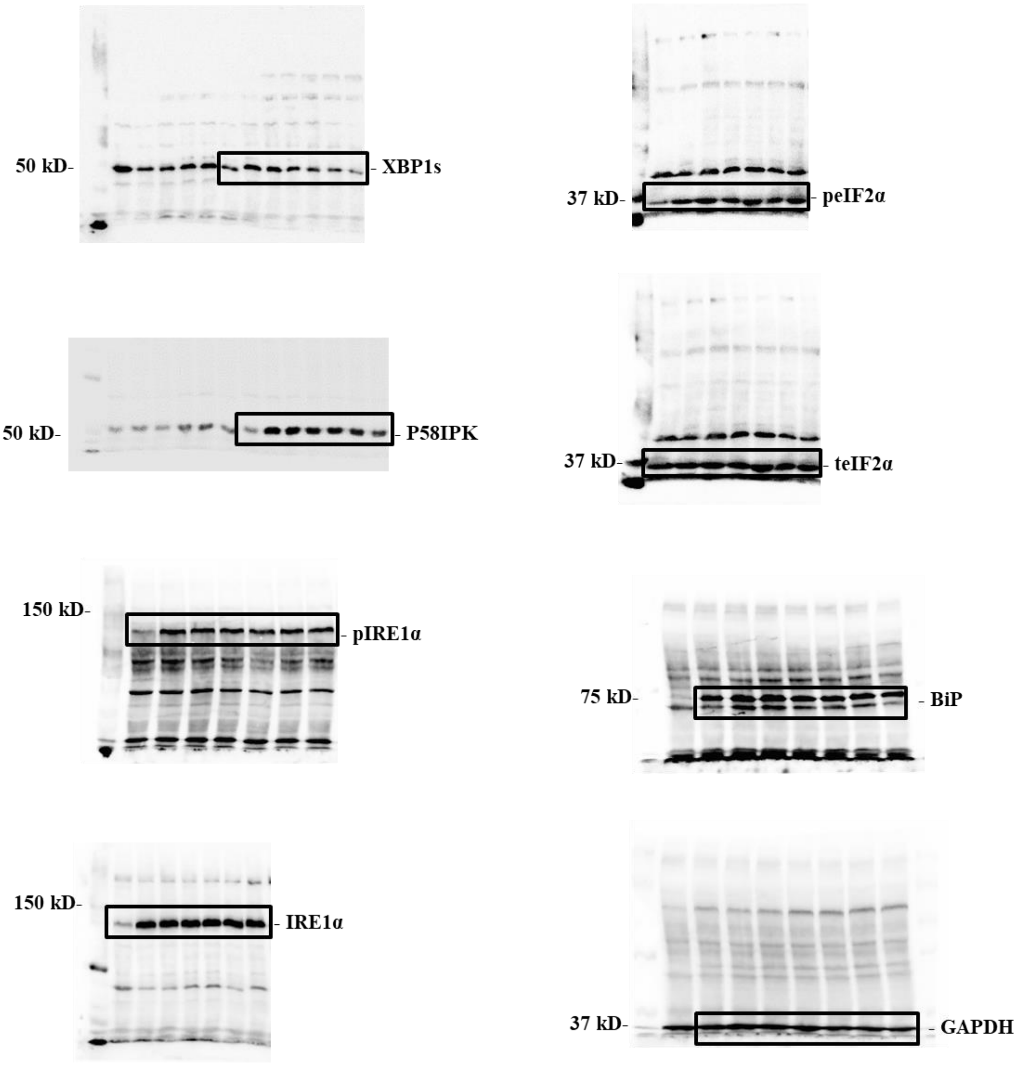

Figure 4e

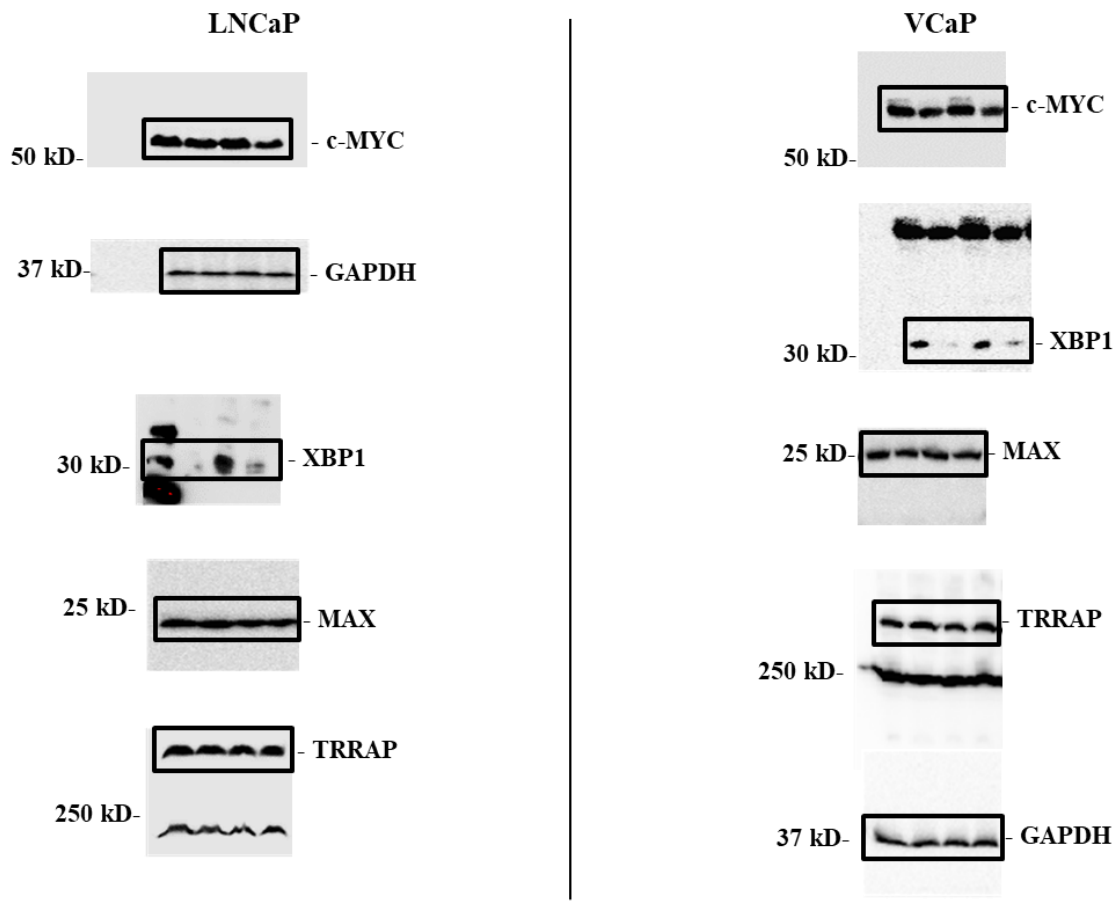

Figure 4f

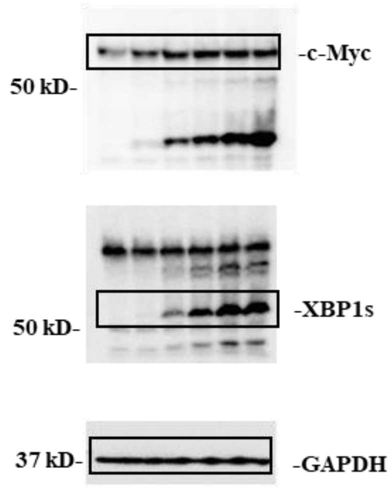

Supplementary Figure 2g.

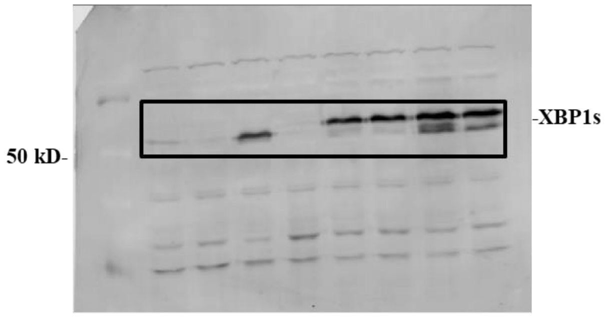

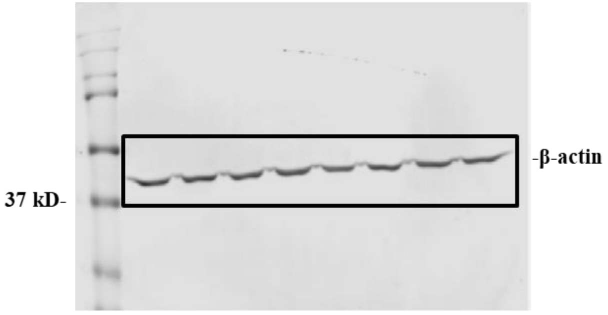

Supplementary Figure 10. Uncropped Western blots of indicated figures.

Supplementary Table 1. qPCR primers used in this study.

Genes	Forward	Reverse
XBP1s	AGTCCGCAGCAGGTGCAG	CTTCCAGCTTGGCTGATGAC
XBP1u	TAGCAGCTCAGACTGCCAGA	ACTGGGTCCAAGTTGTCCAG
EDEM1	GAACCTCCGGAGCAATGATA	GAACCTCCGGAGCAATGATA
RAMP4	CGAGAAGCACAGCAAGAACA	TCACATGCCCATCCTGATAC
P58IPK	AACGCTTTTGGAAGTGGAGA	GCTCTTCAGCTGACTCAATCAG
FICD	CTCAGCATCACCTCCAGG	GTGCATGAAGAGCTTTTGGG
BHLHA15	AGCTAAATAACGCCTTCCAGG	GCGATTTGATGTAGTTCTTGGC
SEC24D	TCACCCTCCCCAAAGATTTC	GGGTGACAGATGAAGTGGATATAG
SERP1	CGCCAAGACCTCGAGAAATG	ACTTCACATGCCCATCCTG
DNAJB9	CATCAGAGCGCCAAATCAAG	TTTCGTCTATTAGCATCTGAGAGTG
FKBP11	ATTCCTTCTCACTTGGCCTATG	TGCCCTTCACCAGCTTTAG
FKBP14	GACGGCTCCTTATTTCACTCC	CTCTTCTCTCCTACACACATTCC
GMPPA	CTACCTCTTTTCTCCTGAAGCC	GCTGAAAACACATCCTGCTC
SYVN1	CTCAAATGTTTCCACTGGCTG	CTGTGATAGGCGTGGCTG
BTN3A1	AAGCAAGAACAAAGCACAAGAG	ATCCAGAATCACATCCGCAG
c-Myc	ACCACCAGCAGCGACTCTGA	TCCAGCAGAAGGTGATCCAGACT
SHMT1	TGCCCTCCCCATTTGAAC	GAGACTCCAGGTTGTACAGAATC
PCNA	CGGATACCTTGGCGCTAGTA	TCACTCCGTCTTTTGCACAG
MTHFD1	CAATCAAGCCCAATCTCATGC	CCGTCACTACAAACCCTTCTG
CDC25A	TGTTGAAGAGACCAGAACGATC	GGGAAGATGCCAGGGATAAAG
ATF4	TCCCATCTCCAGGTGTTCTC	CAGCTCTTTGCACTCACCAG
PPAT	ATGGCGAATTGGTAAATGCTG	GGGTGTCATCTTGTTCCTGAG

MAX	ATGTTTCACTCCTGCTTCTCTC	GCTGCCATCTCCCATACATAC
TRRAP	TGTGGAGGCAGCATCATTAC	GGGATCATGCTGAGAGCTATTC
